# Integrated genomic analysis of triple-negative breast cancers reveals novel microRNAs associated with clinical and molecular phenotypes and sheds light on the pathways they control

**DOI:** 10.1186/1471-2164-14-643

**Published:** 2013-09-23

**Authors:** Emanuele de Rinaldis, Patrycja Gazinska, Anca Mera, Zora Modrusan, Grazyna M Fedorowicz, Brian Burford, Cheryl Gillett, Pierfrancesco Marra, Anita Grigoriadis, David Dornan, Lars Holmberg, Sarah Pinder, Andrew Tutt

**Affiliations:** 1 Breakthrough Breast Cancer Research Unit, Division of Cancer Studies, School of Medicine, King’s College London, Guy’s Hospital, London, UK; 2 NIHR Biomedical Research Centre - R&D Department, Guy’s Hospital, London, United Kingdom; 3 Division of Cancer Studies, School of Medicine, King's College London, Guy’s Hospital, London, UK; 4 Regional Oncologic Centre Uppsala/Örebro, Uppsala, Sweden; 5 Department of Molecular Biology, Genentech, Inc, South San Francisco, CA, USA; 6 Breast Research Pathology, Division of Cancer Studies, School of Medicine, King's College London, Guy’s Hospital, London, UK; 7 Department of Molecular Diagnostics and Cancer Cell Biology, Genentech, Inc., South San Francisco, CA, USA

**Keywords:** miRNAs, Breast cancer, Data integration, Pathway analysis

## Abstract

**Background:**

This study focuses on the analysis of miRNAs expression data in a cohort of 181 well characterised breast cancer samples composed primarily of triple-negative (ER/PR/HER2-negative) tumours with associated genome-wide DNA and mRNA data, extensive patient follow-up and pathological information.

**Results:**

We identified 7 miRNAs associated with prognosis in the triple-negative tumours and an additional 7 when the analysis was extended to the set of all ER-negative cases. miRNAs linked to an unfavourable prognosis were associated with a broad spectrum of motility mechanisms involved in the invasion of stromal tissues, such as cell-adhesion, growth factor-mediated signalling pathways, interaction with the extracellular matrix and cytoskeleton remodelling. When we compared different intrinsic molecular subtypes we found 46 miRNAs that were specifically expressed in one or more intrinsic subtypes. Integrated genomic analyses indicated these miRNAs to be influenced by DNA genomic aberrations and to have an overall influence on the expression levels of their predicted targets. Among others, our analyses highlighted the role of miR-17-92 and miR-106b-25, two polycistronic miRNA clusters with known oncogenic functions. We showed that their basal-like subtype specific up-regulation is influenced by increased DNA copy number and contributes to the transcriptional phenotype as well as the activation of oncogenic pathways in basal-like tumours.

**Conclusions:**

This study analyses previously unreported miRNA, mRNA and DNA data and integrates these with pathological and clinical information, from a well-annotated cohort of breast cancers enriched for triple-negative subtypes. It provides a conceptual framework, as well as integrative methods and system-level results and contributes to elucidate the role of miRNAs as biomarkers and modulators of oncogenic processes in these types of tumours.

## Background

Breast cancer is a heterogeneous disease that comprises tumour subgroups with substantial differences in biology and clinical behaviour. Classification methods based on the expression of intrinsic genes [1] and on the histological assessment of oestrogen- (ER) and progesterone- (PR) receptor and human epidermal growth factor receptor 2 (HER2), have revealed the existence of different subgroups with diverse clinical outcomes and responses to treatment.

Among these, ER-negative and triple-negative breast cancers (ER-/PR-/HER2-negative) are types of aggressive tumours that account for approximately 30% and 15% of breast cancers, respectively [2] and are known to have a poorer prognosis than most ER-positive types. Although various multi-gene prognostic markers have been proposed for the prediction of their clinical outcome, the reliable identification of the small group of patients with ER-negative tumours who have a more favourable prognosis still represents an open challenge (reviewed in [3]).

miRNAs are small non-coding RNA molecules regulating gene expression both at the transcriptional and translational levels. Since 2005, when miRNA deregulation was first described in breast cancer [4], many studies have demonstrated a role for miRNAs in the modulation of oncogenic pathways and their potential as prognostic and/or subtype-specific diagnostic biomarkers (reviewed in [5]). Further interest in miRNAs has stemmed from their demonstrated suitability for analysis in formalin–fixed, paraffin embedded (FFPE) tumour tissues [6].

Until recently, only in a limited number of studies have miRNAs been analysed in the context of transcriptional and genomic profiles obtained from the same breast tumour samples and integrated with clinical-pathological information [7,8]. Even more limited information exists when the focus is restricted to selected tumour sub-cohorts, such as triple-negative breast tumours.

**Figure 1:**
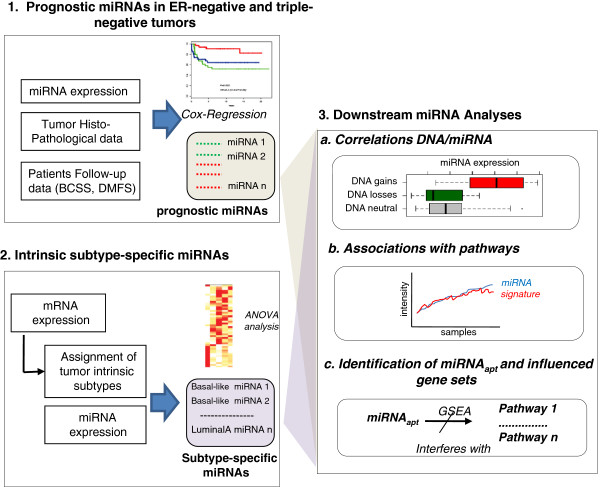
**Overview of the analytical workflow used in the study. 1.** miRNA expression data, histopathological data and patient clinical follow-up information were used in Cox-regression models to identify prognostic miRNAs. **2.** mRNA data was used to classify tumours into different molecular "intrinsic" subytpes. miRNA differential expression across tumour subtypes were then identified using ANOVA analysis. **3.** Prognostic and subtype-specific miRNAs identified in *a* and *b* were further analysed: **(a)** correlations between miRNA DNA copy number aberrations and miRNA expression **(b)** Correlative analysis between miRNA expression and pathway signatures **(c)** Identification of miRNAs anti-correlated with the transcriptional levels of their predicted targets (miRNAapt) and inference of the pathways and transcriptional signatures they control.

Thus, we carried out comprehensive miRNA, mRNA and DNA profiling in a well-annotated cohort of invasive breast cancers (n = 181), concentrating on triple-negative tumours (n = 114). A general overview of the rationale of the study is illustrated in Figure 1. Integrative genomic data analysis, along with clinical and pathological information allowed us to identify prognostic and subtype-specific miRNAs (Figure 1, panels 1-2), to elucidate whether they were affected by DNA genomic aberrations (Figure 1, panel 3a) and to monitor their associative and/or functional relationships with the activity of biological pathways. For the latter point we adopted two independent strategies. The first of these looked at the associative correlations between miRNAs and the level of activation of a large compendium of pathways, inferred from gene-expression signatures (Figure 1, panel 3b). The second strategy aimed to identify the subset of miRNAs potentially playing an effect on the expression levels of their predicated targets and provided a comprehensive description of the pathways and gene sets most significantly influenced by these miRNAs (Figure 1, panel 3c).

By using Cox-regression analysis with distant metastases-free survival (DMFS) and breast cancer specific survival (BCSS) as clinical end points, we found 7 miRNAs associated with prognosis in triple-negative tumours with an additional 7 when the analysis was extended to the larger group of all ER-negative tumours. When we investigated the pathways associated to these miRNA, we found unfavourable prognostic miRNAs to correlate with a broad spectrum of motility mechanisms involved in cell invasion and to growth factor-mediated signalling pathways. To understand the role of miRNAs in the establishment of tumour transcriptional phenotypes we also investigated miRNA expression patterns across different intrinsic molecular subtypes, as defined by the PAM50 classification method [1], and identified 46 subtype-specific miRNAs.

Integrated miRNA-mRNA analyses showed that subtype-specific miRNAs tend to be enriched for anti-correlated genes among their predicted targets. This result represents an exception from the general lack of correlation that was observed between miRNAs and predicted targets expression levels. Among subtype-specific miRNAs, our analysis highlighted the role of miR17-92 and miR-106b-25, two polycistronic miRNA clusters known to play a key oncogenic role in various cancers [9]. These two clusters appeared to be specifically over-expressed in basal-like tumours and their expression to be largely influenced by DNA copy gains. We also observed the anti-correlated predicted targets of miR17-92 and miR-106b-25 miRNAs to be enriched for luminal specific sets of genes. This suggests these miRNAs contribute to the definition of the tumour transcriptional phenotype by influencing the expression levels of these genes.

Interest in the miR17-92 and miR-106b-25 clusters was further strengthened by their inferred interference with known oncogenic processes, including epithelial-mesenchymal transition (EMT), PI3K/AKT/mTOR, MYC and PTEN pathways.

## Results

A schematic representation of the integrated analytical workflow used to generate the presented results is illustrated in Additional file 1: Figure S1.

### Samples data and tumour classification

The study is based on the integrative analysis of miRNA expression, gene expression (GE), and SNP-array based DNA copy numbers (CN) in a set of 181 invasive breast carcinomas extracted from 173 patients (Additional file 2: Figure S2a). Patients were treated at Guy’s and St Thomas’ Hospitals, London, UK between 1979 and 2001 and had at least 5.5 years follow-up. Of the 181 tumours analysed, 123 were immunohistochemically ER-negative, 114 being also triple-negative (ER-, PR- and HER2-negative). Molecular characteristics of tumour samples were analysed in association with clinical and pathological information (Additional file 3: Table S1). In addition to the assignment to clinical subgroups based on ER, PR and HER2 status, 142 tumour samples were also assigned to the five intrinsic molecular subtypes (basal-like, luminal A, luminal B, HER2 and normal-like) using the expression of predefined intrinsic gene lists according to the PAM50 centroid-based classification method [1,10]. In agreement with previous studies [11], triple-negative breast cancers were found to correspond mostly with the basal-like tumours (87%) while ER-positive lesions corresponded to luminal A and B subtypes (87%) (Additional file 2: Figure S2b).

### miRNA copy number and expression

miRNAs are frequently located in regions of genomic instability [12], and miRNA expression changes have been found to be associated with chromosomal rearrangements in many tumours, including breast cancer [12,13]. In order to gain a general view on the impact of DNA aberrations on miRNA expression in this cohort, Spearman correlation coefficients between DNA copy number of miRNA loci (CN) and miRNA expression values were computed. In addition, for each miRNA DNA locus identified as altered in any of the samples, we performed separate non-parametric Wilcoxon rank sum tests to assess differences in expression between samples with losses and gains, compared to samples with no copy number alterations. miRNAs co-located with transposable elements (as reported in [14]) – given their uncertain genomic location were excluded from this analysis.

As a result we identified 64 miRNAs showing statistically significant miRNA-CN correlation, indicating an overall influence of genetic aberrations (gains and losses) on the expression of the miRNAs (Additional file 4: Table S2). 17/64 of these miRNAs were identified to fall into breast cancer recurrent aberrant regions [8]. Of these, respectively 11 and 6 miRNAs fall into regions of focal recurrent amplifications and focal recurrent deletions (see details in Methods and Additional file 4: Table S2). These results suggest the expression values of these miRNAs to be frequently perturbed in breast cancers, as result of underlying DNA aberrations.

We have then carried out an analysis to assess the likelihood that copy-number driven miRNA could be co-amplified/co-deleted with key cancer related genes. For each copy-number driven miRNA, a region spanning 10 Kilobases before and after its genomic location was isolated and oncogenes and tumor suppressor genes according to the "Cancer Genes DB" [15] were isolated. This resulted in a list of co-amplified/co-deleted tumour suppressor and/or oncogenes for each miRNA (Additional file 4: Table S2).

As examples, we found miR-93 and miR-106b to be co-amplified with PI3K and MET and miR-548 to be co-amplified with MYC, suggesting a (functional or just correlative) relationship between the expression levels of these miRNAs and the activities of PI3K/AKT, MYC and MET pathways.

### Expression analysis of miRNA and candidate target transcripts

To assess the relationships between miRNAs and their target genes, we carried out a correlation analysis between each miRNA and the expression levels of their respective predicted transcripts. The rationale for this analysis was based on three points: i) predicted targets are largely unreliable and therefore cannot be directly used to derive any sound biological observation ii) among all predicted miRNA targets, the real ones - those transcriptionally degraded upon miRNA binding - are anti-correlated to their cognate miRNA (whilst this is a required condition, it does not prove a predicted target to be real) iii) miRNAs showing an enrichment among their predicted targets for anti-correlated transcripts, are more likely to play a role in their control.

For each miRNA, lists of candidate targets were extracted using six different prediction algorithms (see Methods) and independent analyses were run on each list. Our data indicated the general lack of correlation between miRNAs and predicted targeted mRNAs, irrespective of the algorithm used for target prediction (Additional file 5: Figure S3). We focused then on the identification of individual miRNAs deviating from this general behaviour, i.e. for which a general anti-correlation with the expression levels of their predicted targeted mRNAs could be detected. We labelled these entities as miRNAapt ("miRNA anti-correlated to predicted targets") and devised a strategy for their identification based on the evaluation of their bias towards the enrichment for anti-correlated genes among their predicted targets (see Methods). By using stringent cut-offs we have identified a total of 43 miRNAapt (Additional file 6: Table S3 and Additional file 7: Figure S4).

### miRNAs associated with prognosis in ER-negative and triple-negative breast cancers

Cox-regression univariate analysis was carried out to identify miRNAs whose expression was associated with clinical outcome. Analyses were run separately for the ER-negative and the sub-set of triple-negative tumours, with respect to breast cancer specific survival (BCSS) and distant metastases-free survival (DMFS) clinical end points.

**Figure 2:**
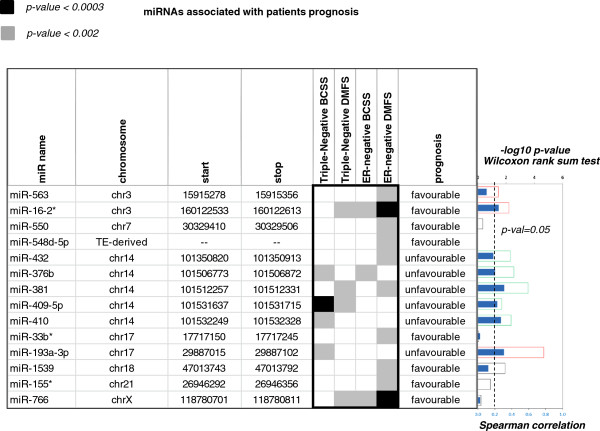
**Prognostic miRNAs in ER-negative and triple-negative tumour sub-cohorts (log-rank p-value < 0.002 and FDR q-value < 0.2).** Chromosomal positions are indicated, except from miR-548d-5p, which co-locates with a DNA transposable element (TE). **Left Panel**: Results from independent Cox-regression analyses run on triple-negative and ER-negative tumours **Right Panel**: Association between miRNA expression and DNA gains/losses. Filled blue bars represent Spearman correlation values. Shaded bars represent the -log10 q-value of the Wilcoxon test, resulting from the comparisons of tumours with DNA gains (red bars) and losses (green bars) to tumours without DNA changes.

**Figure 3:**
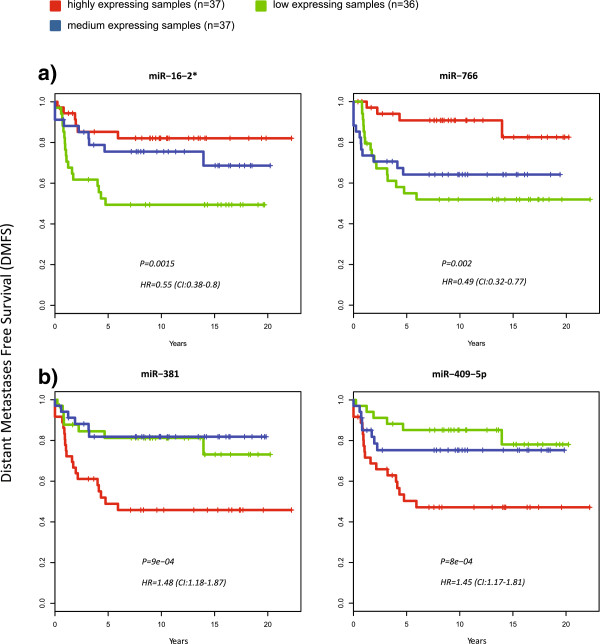
**Kaplan-Meier curves of the four miRNAs showing strongest association with distant-metastases free survival (DMFS) in triple-negative tumours.** Patients are stratified into three groups according to the miRNA expression level tertiles. **(a)** "favourable" miRNAs, whose expression is associated with better prognosis (HR < 1) **(b)** "unfavourable" miRNAs, whose expression is associated with poorer prognosis (HR < 1). The hazard ratio indicates the risk change if miRNA expression rises by one standard deviation.

As result, 14 miRNAs associated with prognosis were identified in ER-negative tumours; of these 7 were also significant in the sub-set of triple-negative tumours (p-value < 0.002, FDR < 0.2) (Figures 2 and 3, Additional file 8: Figures S5 and S6).

miRNA-DNA analyses showed miR-193a-3p to have the strongest association with DNA copy numbers, with DNA gains being associated with miRNA over-expression. Prognostic miRNAs tended to be sparsely distributed in the genome, with the exception of a cluster of miRNAs located in close proximity to one another on band 14q32.31, comprising miR-376b/miR-381/miR-409-5p/miR-410, all linked to unfavourable prognosis. We found, as have others, that this cluster is subject to frequent genomic deletions [16], causing decrease in expression of miR-376b and miR-381 (Figure 3).

When univariate and multivariate analyses were run using histopathological information on its own (without miRNA expression), node positivity, tumour size and percentage of tumour lymphocytic infiltration emerged as statistically significant prognostic factors (Additional file 8 and Additional file 9: Table S4). We then assessed how these covariates impacted miRNA association with prognosis, by evaluating additive multivariate Cox-regression models. P-value distributions derived from different models were compared, showing an impact of the percentage of lymphocytic infiltration on the association of miRNAs with prognosis (Additional file 8: Figure S7). miRNAs were shown to be less informative with respect to prognosis when evaluated in multivariate models incorporating information on lymphocytic infiltration.

Among the 14 miRNAs collectively identified to be associated with prognosis alone, five retained their prognostic value when evaluated in models which included node positivity and tumour size (miR-376b, miR-381, miR-409-5p, miR-410, and miR-766) and only one (miR-193a-3p) when lymphocytic infiltration was also considered, either in TNBC or in the wider group of ER-negative samples (including TNBC samples) or both (Additional file 8: Figure S8).

We have then analysed two independent miRNA data sets: one from Buffa et al., including 82 ER-negative and 37 triple-negative breast cancer samples [17]; the second from Enerly et al, including 32 ER-negative and 21 triple-negative samples [18]. All analyses were run independently in the ER-negative group and its subset of and triple-negative tumours.

Analyses showed broad lack of reproducibility between the three sample cohorts with regard to miRNA associations with prognosis (Additional file 8: Figures S9 and S10). Furthermore, direct comparison between our results and the recently published results from the study of Dvinge et al. colleagues, again did not show overlap [7]. As discussed more extensively below, the different sample size of the studies as well as differences in the cohort demographics (we selected patients who had not have received neo-adjuvant treatment to avoid biases due to pre-surgery treatment while the other studies considered mixed populations) and the potential for differing representations of histopathological characteristics and biological subgroups within heterogeneous triple negative breast cancer, are all factors that might account for the general lack of reproducibility of analytical results with regard to the prognostic impact of miRNAs.

Finally, we have assessed how miRNAs compare with mRNAs with respect to association with BCSS and DMFS. We run Cox-regression survival analysis on TNBC and ER samples using gene expression and miRNA data separately, without including histopathological information in the model. When we compared the distributions of p-values, no significant differences between the two data sets emerged (Additional file 8: Figure S11), indicating an overall comparable association of miRNA and mRNA to ER-negative and TNBC patients prognosis.

### Pathways associated with prognostic miRNAs in ER-negative and triple-negative tumours

To gain insights into the biological role of the identified prognostic miRNAs, we explored the relationships between their expression and a large compendium of pathways, biological processes and molecular functions represented by gene sets extracted from public (KEGG [19], Panther [20]) and commercial (GeneGO [21] and Ingenuity [22]) databases. The analysis was run using gene expression data to compute a "gene set score" for each gene set in each sample (see Methods). Correlations between miRNA expression levels and gene set scores across all tumour samples were calculated and used for unsupervised hierarchical clustering analysis. In this way similarities between miRNAs associated to the pathways represented by their respective gene sets could be highlighted.

**Figure 4:**
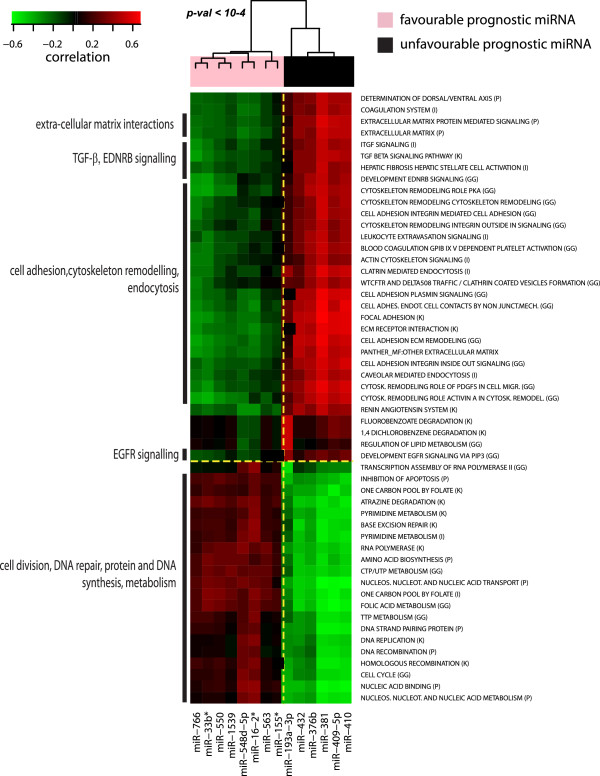
**Pathways associated to the expression of prognostic miRNAs.** 2D Hierarchical Clustering heat map using Spearman correlations between prognostic miRNAs in TNBC and ER-negative breast tumours (heat map columns) and gene set scores representing pathways activation (heat map rows). Gene set sources are indicated in brackets: GG (GeneGo), I (Ingenuity), K (KEGG), P (Panther). The p-value on the dendogram indicates the statistical significance of the clusters, evaluated using bootstrapping analysis (n = 10.000).

As a result, a clear separation between unfavourable/favourable miRNAs was obtained (bootstrapping p-value < 10-4), indicating that their opposite prognostic values are reflected in the association with different classes of pathways (Figure 4). Many of the biological themes positively correlated with unfavourable prognostic miRNAs are linked to cell motility, a critical step in the promotion of cancer invasion and metastasis, and to the broad spectrum of motility-related mechanisms such as cell adhesion, interaction with the extracellular matrix, cytoskeleton remodelling and control of membrane proteins through clathrin-mediated endocytosis. Others relate to intracellular signalling pathways, such as those activated by growth factors (TGF-β and the EGFR signalling pathways). We also found a positive correlation with the renin-angiotensin system, a process reported to be involved in regulation of tumour angiogenesis in ER-negative breast cancers [23]. Conversely, favourable prognostic miRNAs were found to be associated with activities linked to proliferation such as enhanced cell division, inhibition of apoptosis, DNA repair, protein and DNA synthesis and metabolism.

### Subtype-specific miRNAs

To understand the role of miRNAs in the establishment of tumour transcriptional phenotypes we also investigated miRNA expression patterns across different intrinsic molecular subtypes, as defined by the PAM50 classification method [1]. To this aim we carried out univariate ANOVA analysis and identified 46 miRNAs differentially expressed across the five intrinsic subtypes (q-value < 0.001). Of these, 13 showed highest expression in basal-like, 23 in luminal tumours (A or B), 6 in normal-like and 4 in HER2 tumours (Additional file 8: Figure S12 and Additional file 4: Table S2). One of them (miR-193a-3p) was also prognostic in triple-negative tumours. When we compared the list of specific subtype miRNAs with that of the miRNAs influenced by DNA copy number changes we found 15 miRNAs shared between the two groups, representing a statistically significant overlap (Fisher test p-value < 0.001). This data indicates the tendency of specific subtype miRNAs to be regulated at the genetic level, by an altered number of copies. Results on subtype-specific miRNAs are consistent with those reported from independent studies [7,24] (Additional file 8: Figure S16). Notably, among the miRNAs most up-regulated in basal-like tumours we observed members of the miR-17-92 cluster (miR-20a, miR-92a, miR-17/*, miR-19a/b, miR-18a) and its paralog miR-106b-25 (miR-106b, miR-93). These two miRNA clusters have been reported to be amplified and/or over-expressed in a variety of hematopoietic and solid tumours, including breast [25], and are emerging as key modulators of various cancer –associated processes, including proliferation, apoptosis and angiogenesis (reviewed in [26,27]).

### Analysis of gene sets potentially influenced by individual miRNAapt

We isolated for each miRNAapt the list of genes predicted to be targeted by prediction algorithms and, at the same time, being anti-correlated to the miRNAapt, assuming these lists to be enriched for real targets, as illustrated above.

Running a gene set enrichment analysis on these targets-enriched lists we could then establish a link between miRNAapt and the gene sets potentially influenced by their action (Additional file 7: Figure S4).

For the identification of the pathways and transcriptional signatures influenced by miRNAapt we used an adaptation of the approach described by Creighton and colleagues [28], based on gene set enrichment analysis of miRNAs predicted targets through Fisher’s exact test. The algorithm was modified to use for each miRNA only the list of anti-correlated predicted targets, and to run against a comprehensive compendium of published gene sets, representing published transcriptional signatures (the *Molecular Signatures Database*) [29] as well pathways, biological processes and molecular functions extracted from public (KEGG [19], Panther [20]) and commercial (GeneGO [21] and Ingenuity [22] databases.

In this way a link between each miRNAapt and the gene sets potentially influenced by its suppressive action could be established.

As a result, for the 43 identified miRNAapt the analysis retrieved a total of more than 200 gene sets (using ~4,000 gene sets as input) that were significantly associated to one or more miRNAapt. The complete set of results is reported in Additional file 6: Table S3.

The whole procedure was verified using external data on 11 miRNAapt, from an independent study by Linsley et al. [30]. For these miRNAs, the effects on the predicted targets had been experimentally determined using miRNA transfection experiments in *in-vitro* cancer cells, followed by gene expression analysis by microarrays. In this way, they had derived for each miRNA the list of down-regulated targeted transcripts in *in-vitro* cell lines.

We observed that these mRNA gene sets were correctly predicted by our algorithm as being respectively influenced by the 11 miRNAapt tested. In fact, for each of these miRNAapt, the experimentally validated list of its target genes showed up as the most significantly enriched gene set (p-value < 10^-8^, Additional file 10: Figure S18), thus providing direct evidence of the validity of the method. Moreover, the procedure has been extended based on miRNA validated targets reported in the miRTarBase [9]. For miRNAs for which significant information on validated targets was available, such as let7-b, miR-17 and miR-29c, we could confirm many of our inferences on their targeted pathways. Examples are miR-17 association with PI3K-AKT and TGF signalling pathways, let7-b association with PI3K and miR-29c association with PTEN pathways (Additional file 4: Table S2), discussed more extensively below.

### Transcriptional signatures and pathways potentially impacted by the action of subtype-specific miRNAs

**Figure 5:**
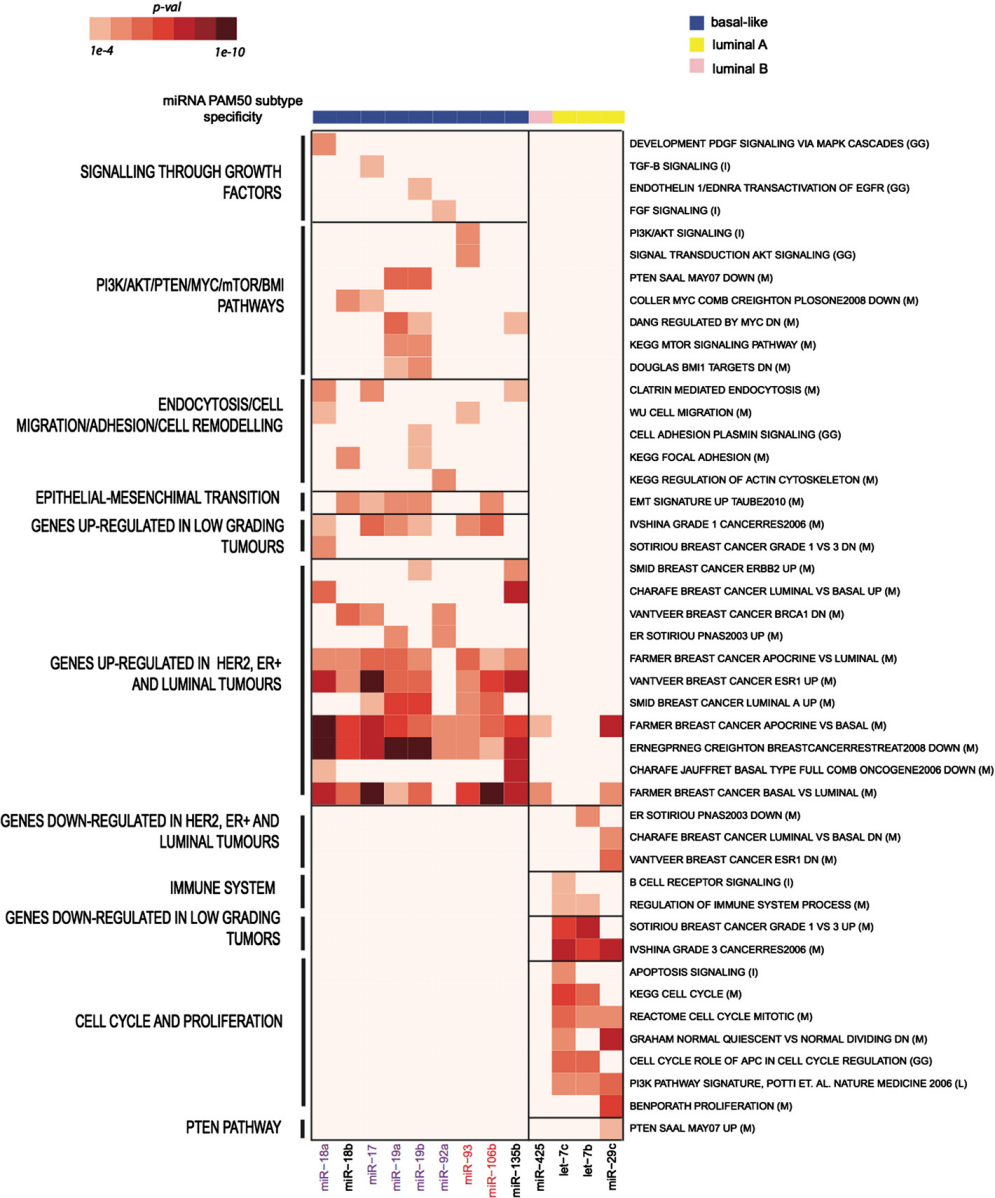
**Pathways and signatures potentially influenced by the action of miRNAapt.** For each miRNAapt (columns), the heatmap represents gene set enrichments (expressed as the -log10 of the Fisher-test p-value), in the list of individual anti-correlated miRNAapt target genes. miR-106b-25 and miR-17-92 cluster members are respectively highlighted in red and purple. Gene set sources are indicated in brackets: GG (GeneGo), I (Ingenuity), K (KEGG), P (Panther), L (Literature).

Of the 46 subtype-specific miRNAs, 14 were classified as miRNAapt suggesting a potential role of these miRNAs in influencing the expression levels their targets (Fisher test p-value < 10e-5). Among these are miRNAs of the miR-17-92 (miR-17, miR-20a, mir-18a, miR-19a/19b, and miR-92a) and miR-106b-25 (miR-93, miR-106b) clusters, all up-regulated in the basal-like subtype of breast cancer. These miRNAs were associated to gene sets reported to be over-expressed in luminal and ER-positive tumours or over-expressed in low-grade tumours, in independent studies (Figure 5).

In addition, miR-19b links to a gene set that is up-regulated in the HER2 positive subtype of the disease.

We also observed the association with cancer related gene sets, such as the MYC down-regulated gene set (miR-17 and miR-18b), as well as gene sets representing mTOR and PTEN pathways (miR-19a/b). Other gene sets associated to miR-17-92 cluster include those related to tumour proliferation, such as the PDGF (miR-18a), TGF-β (miR-17) and FGF (miR-92a) pathways, as well as gene sets involved in cell migration (miR-18a) and endocytosis (miR-17, miR-18a). Furthermore, we observed the association of an epithelial-mesenchymal transition transcriptional signature by miR-17, miR-19a/b and miR-106b. miR-19b is also linked to elements of focal adhesion and endothelium, while miR-92a is involved with the regulation of cytoskeleton.

When we looked at luminal A and B specific miRNAs, we found that let-7b/c and miR-29c link to gene sets that were down-regulated in luminal and ER-positive tumours and up-regulated in basal-like and ER-negative tumours. Cell cycle, proliferation and tumour grading gene sets are also found to be associated with let-7b/c, consistently with their reported role of as tumour suppressors, functioning as inhibitors of the cell cycle and regulators of apoptosis [31]. Interestingly, additional gene sets influenced by let-7b/c relate to the regulation of the immune system, in keeping with the proposed tumour suppressor role of let-7 [32].

In order to assess the validity of our findings on the functional role of identified subtype-specific miRNAapt we have compared our results with experimental ones, derived from published independent *in-vitro* and *in-vivo* functional studies by others on miR-17-92 and miR-106b-25 miRNAs (Additional file 8). Many of these experiments confirmed our proposed relationships between miRNAs and the gene sets and pathways influenced by their action. These include experiments on miR-17-92 and miR-106b-25 clusters.

## Discussion

We present the results of the integrated analysis of miRNA, mRNA and DNA data from a large breast cancer cohort strongly enriched for triple-negative types and extensively annotated with clinical and pathological information. The work spans multiple lines of analysis, aiming to elucidate different aspects of miRNA breast cancer biology.

### What are the links between genomics aberrations, miRNA expression levels and transcriptional down-regulation of target genes?

We found the expression levels of 64 miRNAs to be statistically influenced by DNA copy number changes. This behaviour effects in particular sub-type specific miRNAs and supports the notion that copy number alterations of miRNAs account partly for miRNA gene deregulation [13]. Of these 64, respectively 11 and 6 miRNAs fall into regions of focal recurrent amplifications and focal recurrent deletions, pointing towards a frequent expression perturbation of these miRNAs as result of underlying DNA aberrations.

As tumours harbour a great amount of transcriptional alterations it is difficult to distinguish "causes" - occurred upstream in the process of tumour formation - from downstream "effects" events. The identification of genetically driven miRNAs helps highlighting those miRNAs that have been altered upstream in the chain of deregulation events, therefore being more likely to play a role in the establishment and maintenance of the tumour phenotype.

With respect to the relationships between miRNA and cognate targets, recent evidence indicates that miRNAs influence gene expression mainly by causing degradation of their target mRNAs, and only to a smaller extent by inhibiting protein translation [33,34]. However, in line with previous miRNA-mRNA analyses of breast tumours, as well as other settings [35,36], our data showed a general lack of correlation between miRNA expression and predicted mRNA targets. This is possibly due to the large presence of false positives in the list of predicted targets. An additional explanation can be that the effects exerted by miRNAs on their targets are limited to a subset of miRNAs, rather than being broadly extended to all of them.

By means of a specific analytical workflow we identified 43 of these miRNAs - that we named miRNAapt. We then established functional links to the pathways and gene signatures on which miRNAapt are likely to exert a negative control.

### Do miRNAs hold potential as prognostic markers in ER-negative and triple-negative breast cancer?

We have identified 14 miRNAs associated with clinical outcome in the set of all ER-negative cancers; 7 of these were associated with prognosis also in the sub-group of triple-negative tumours. We have then endeavoured to shed light on the pathways associated to the expression levels of each of these prognostic miRNAs. Our analysis did not allow us to distinguish between associative and causal relationships. However, the correlation of unfavourable prognostic miRNAs with a number of cancer related pathways provides indirect (and independent) evidence of their association with the biology of the tumours. In fact, among the pathways correlated with unfavourable miRNAs, we found a recurrent emergence of cell motility and related mechanisms - processes that are known to facilitate the invasion of surrounding stromal tissues [37,38].

Other emerging themes associated with unfavourable miRNAs were those related to TGF-β and EGFR intracellular signalling pathways – known to play a pivotal role in metastasis [37,38] – and to the stromal receptor *EDNRB* – which is described as being involved in the acquisition of invasive potential of pre-malignant breast lesions [39]. Surprisingly, favourable miRNAs were instead found to be positively associated with cell activities related to proliferation. A possible explanation for this association is that tumour proliferation, as inferred from transcriptional data, could mirror the enhanced engagement and/or proliferation of immune cells (B- and T-lymphoid cells), thus representing a protective factor.

A positive association between a proliferation metagene, a B cell metagene and good outcome in breast cancers has been observed by others in independent data sets (TRANSBIG breast cancer cohort, [40]). Further indications of the puzzling relationship between proliferation and patient survival in triple-negative breast cancers come also from recent studies, reporting the lack of association between standard markers of proliferation and prognosis in these types of tumours [41,42].

When we compared our results with those obtained from independent data sets [7,17,18], no overlaps were found. These results point towards the elusive nature of miRNAs when endeavours are made to validate their prognostic associations across independent studies as also previously highlighted [7]. Noticeably, lack of consistency also emerged when the three external studies were compared between themselves. Beyond the different powers deriving from different sample sizes of the studies, other factors may account for the observed inter-study differences. Among these are the cohort demographics (we selected patients who had not have received neo-adjuvant treatment to avoid biases due to pre-surgery treatment while the other studies considered mixed populations). Another element which might account for inter-study differences is the impact of histopathological characteristics on the association of miRNAs expression with the clinical follow-up - in particular the level of lymphocytic infiltration, as we have observed in our data. Thus, reported associations of miRNA expression levels with prognosis might mirror - at least in part - different degrees of tumour lymphocytic infiltration, more than miRNA specific processes occurring in tumour epithelial cells. In fact, even though stromal cells are only a minority of the whole tumour sample, miRNAs from both epithelial and stromal compartments will have been profiled and contributed to associations observed. A related confounding factor is the inherent heterogeneity of triple-negative breast cancer [43], whose subclasses might be differentially represented across different patient data sets.

These aspects are particularly critical in consideration that the great majority of miRNAs are only weakly associated with prognosis, and point towards the need to take them into account when molecular-prognostic studies are carried out. Further experimental efforts are needed to shed light on the functional role of each of these miRNAs in the triple-negative breast cancer setting. By complementing top-down statistical approaches with the acquisition of functional information it will be possible to understand the biological mechanisms underpinning the link of a given miRNA to prognosis, and possibly allowing dissection of the reasons why this link can be lost in different patient cohorts. The pathway correlation analysis we have carried out for the 14 prognosis associated miRNAs represents an endeavour in this direction. However, whilst internal cross-validation checks of our results with the results from pathway analysis together provide an indication of their statistical and biological significance, further functional validation is warranted.

### How are miRNAs expressed in different intrinsic tumour subtypes?

We found 46 miRNAs differentially expressed across different intrinsic tumour subtypes, 13 and 23 respectively showing the highest expression in basal-like and luminal A and B tumours (Additional file 8: Figure S12 and Additional file 4: Table S2). As anticipated from the known relationships between the different classification schemes and pathological tumour features, miRNAs associated with the basal-like intrinsic subtype tend to be also over-expressed in triple-negative and high-grade tumours. The comparison of our results with equivalent analyses run in independent studies [7,24] showed a very good level of agreement, therefore confirming their validity (Additional file 8: Figure S15). Of interest, the link between breast cancer PAM50 subtypes and miRNA expression was also recently reinforced by the extensive analysis run by The Cancer Genome Atlas Network (TCGA) [8]. Here the authors presented an integrated genomic analysis of 501 breast cancer samples, classified on the basis of mRNA expression - using the same PAM50 classification criteria we have used - as well as miRNA expression. They demonstrated a strong convergence between the miRNA- and mRNA (PAM50)-based classification systems, pointing towards the fact that the PAM50 subtype classification is reflected, at the miRNA level, into the subtype-specific expression of a large number of miRNAs.

We found the expression levels of 15 of the 46 subtype specific miRNAs to be significantly associated with DNA copy number changes, indicating a large fraction of miRNA expression differences between different molecular subtypes can be ascribed to upstream genetic tumour aberrations. These include the miR-17-92 and the miR-106b-25 miRNA clusters, highly up-regulated in the basal-like tumours in this series and reported to be amplified and over-expressed in various tumours. These clusters also function as modulators of different cancer processes, including proliferation, apoptosis and angiogenesis (reviewed in [26,27]).

### What is the role of subtype-specific miRNAs in the establishment of different tumour transcriptional phenotypes?

**Figure 6:**
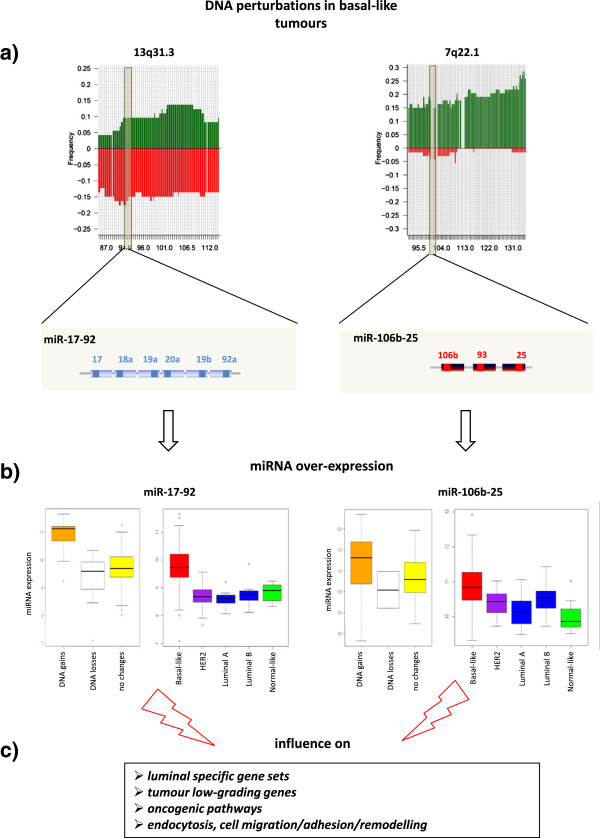
**Summary of the integrated genomic analysis of miR-17-92 and miR-106b-25 clusters. (a)** DNA gains and losses respectively occurring in the miRNA clusters genomic locations. Frequencies of DNA gains (Y axis, green colour) and losses (Y axis, red colour) along the genome sequence (X axis) are represented. Data refer to the selected sub-cohort of basal-like tumours. **(b)** DNA gains in genomic regions 13q31.3 and 7q22.1 drive the up-regulation of the respective miRNA clusters in basal-like tumours. The two paired panels show miRNA cluster expression across tumours with DNA gains (orange), losses (white), no changes (yellow) of the respective loci (left) and different intrinsic subtypes (right) **(c)** down-stream effects observed to be driven, at the transcriptional level, by the up-regulation miR-17-92 and miR-106b-25 clusters in basal-like tumours.

Out of the total of 46 subtype-specific miRNAs, 14 were also identified to function as miRNAapt, suggesting a tendency of subtype-specific miRNAs to influence the expression levels of their targets. These included the basal-like breast cancer specific clusters miR-17-92 (miR-17, 18a, 19a, 19b, 92a) and miR-106b-25 (miR-106b, miR-93), and the luminal breast cancer specific miR-425 (luminal B), let-7b/c and miR-29c (luminal A). Clusters miR-17-92 and miR-106b-25 were shown to impact on transcriptional signatures reported to be up-regulated in ER-positive, luminal tumours or down-regulated in ER-negative basal-like tumours (Figure 6).

These findings open up a new perspective on the interpretation of these signatures, supporting the hypothesis that they can be significantly influenced by the action of mi-17-92 and miR-106b-25 clusters. By also taking in consideration the miRNA-DNA and miRNA-mRNA associations previously discussed, a picture could be delineated in which genomic aberrations could contribute to the establishment of the transcriptional phenotype of basal-like tumours by altering the expression levels of miR-17-92 and miR-106b-25 miRNAs (along with that of their respective host genes c13orf25 and MCM7) which, in turn, decrease the transcript levels of luminal-specific gene signatures. It should be noted that the detected DNA losses of the miR-17-92 region seem to affect the expression of the miRNA cluster only to a limited, and not statistically significant, degree (Figure 6, Additional file 8: Figure S15). Conversely, luminal A specific miRNAs (let-7b/c and miR-29c) were found to target gene sets down-regulated in luminal and ER-positive tumours.

### What is the role of subtype-specific miRNAs in the regulation of oncogenic pathways?

We have identified and described a large number of cancer-related pathways potentially influenced by the action of miRNAapt. These include the MYC, mTOR, TGF-β and PTEN and AKT pathways as well as a transcriptional signature of EMT.

Our findings were supported by published experimental evidences of others (Additional file 8), altogether pointing towards the plausibility of the inferences generated by our analytical procedure on the gene sets influenced by subtype-specific miRNAapt. Some of our findings could also be confirmed when only experimentally validated targets were used for the analysis, as extracted from the miRTarBase database [9]. Examples are miR-17 association with PI3K-AKT and TGF signalling pathways and let7-b association with PI3K and miR-29c association with PTEN pathways (Additional file 4: Table S2).

Analyses of cancer genes co-amplified/co-deleted with miRNAs indicate that some of the identified links between these two miRNAs and oncogenic pathways could be due to associative rather causal relationships. Examples are miR-93 and miR-106b, co-amplified with PI3K and MET and miR-548 co-amplified with MYC.

Whilst a conclusive proof of our analytical findings will have to await functional experiments in basal-like breast cancer models, we believe these pieces of evidence provide indication of the overall validity of our analyses.

## Conclusions

In this study we analysed miRNA, mRNA and DNA data integrated with pathological and clinical information in a large and well-characterised cohort of triple-negative breast cancers. The results presented advance current knowledge of the role of miRNAs as modulators of oncogenic processes in these types of tumours, shedding light on their function in the control of subtype-specific transcriptional signatures and potential as biomarkers. Moreover, this study provides a conceptual framework, as well as integrative methods and system-level results to dissect the relationships between DNA genomic aberrations and down-stream perturbations of miRNAs, target genes and biological pathways.

## Methods

### Patient characteristics

This study considered a retrospective series of 173 patients with early primary breast cancer, treated at Guy’s and St Thomas’ Hospitals, London, UK between 1979 and 2001. The end of study was fixed at January 2007, insuring all patients had at least 5.5 years follow-up. Ethical approval for the analysis of tissue samples and patient notes was obtained from the local research ethics committee and in accordance with the ethical principles expressed in the Declaration of Helsinki. Written informed consent for participation in the study was obtained from all participants. Access to the pseudo-anonymised samples (and clinical data) was obtained in accordance with the principles of the Guy’s & St Thomas Research Tissue & Data Bank (REC No 07/H0804/131). (Guy’s Research Ethics Committee. Ref. 07/H0804/131 Approved on 19/03/2008).

After surgery, patients received adjuvant chemotherapy, adjuvant hormone therapy, adjuvant radiotherapy or a combination of these (details of treatment for each patient are available in Additional file 3: Table S1).

The clinical endpoints considered were distant metastases-free survival (DMFS) and breast cancer specific survival (BCSS).

### Sample preparation

An H&E stained section was cut from each sample of frozen tissue to assess the presence of invasive tumour, to determine cellularity and as a guide for needle micro-dissection. Subsequent frozen sections were stained with Nuclear Fast Red (NFR) and needle micro-dissected removing all normal or non-invasive tissue. The remaining micro-dissected regions with greatest density of invasive tumour cells were selected for nucleic acid extraction. RNA and DNA were extracted using the miRNeasy Mini (Cat. No. 217004) and DNeasy Blood & Tissue (Cat. No. 69504) Qiagen kits respectively, using the manufacturer’s protocols. The quantity and purity of nucleic acids were assessed using Thermo Scientific NanoDrop™ 1000, which generated spectrophotometric analysis of the RNA and DNA concentrations (ng/μl), and 260/280 and 260/230 ratios for each sample.

Based on spectrophotometric analysis, samples identified with any obvious contamination were purified using modified Qiagen protocols. RNA integrity was assessed by using the 2100 Bioanalyzer system, with the RNA integrity number (RIN) obtained using Agilent RNA 6000 Nano Kit (Cat. No. 5067-1511). DNA quality was assessed using 1% agarose gels. Minimum RNA quality requirements for genomic profiling were: i) 60 ng/μl in 10 μl total volume ii) 260/280 ratio ~2, 260/230 ratio > 1.5 and iii) Bioanalyzer (RIN number) > 5. Minimum DNA quality requirements were: i) 50 ng/μl in 30 μl total volume and ii) 260/280 ratio ~2. DNA, miRNA and mRNA profiles were obtained by using Agilent Human 8x15k miRNA arrays, (based on Sanger miRBase release 12.0, containing probes for 866 human and 89 human viral miRNAs), Affymetrix Exon 1.0 ST arrays, and Affymetrix SNP 6.0 arrays, respectively. Standard manufacturer protocols were followed for the amplification, hybridisation, washing, and scanning of the samples.

### Tumour classification

The study was conducted using for each tumour, two mirror samples respectively frozen and formalin fixed and paraffin embedded (FFPE). The frozen samples were used for nucleic acids extraction and the FFPEs were assayed by IHC and CISH using Tissue Microarrays (TMA).

All the tumours were histologically classified using the Guidelines for Pathology Reporting in Breast Cancer Screening approved by the NHS Breast Screening Programme (NHSBSP) and The Royal College of Pathologists (RCPath) NHSBSP Publication No 58 January 2005.

Representative areas of the invasive FFPE tumours were marked for TMA core extraction. TMAs were made in triplicate from the tumour representative areas and 3 μm sections were cut for immunohistochemistry. The expression of ER, PR and HER2 was re-assessed on the TMAs and compared with original receptor data used for clinical decisions. Immunohistochemical demonstration of ER (clone SP-1), PR (clone 1A6) and HER2 was carried out using the automated Leica BOND-Max system. HER2 was also repeated on the automated Ventana system HER2 module using the proprietary ready to use FDA approved kit. In addition, HER2 (2+ cases) were assessed using Dual-chromagen/Dual-hapten In-situ hybridisation (DDISH) staining on the Ventana system. ER and PR status were considered positive if the average Allred score for triplicate cores was more than 2 [44]. If any of the triplicate TMAs had a score of 3+ or 2+ with a HER2 CISH ratio of >2.0 the case was considered to have a HER2 positive status [45]. Additional details of receptor demonstration and evaluation are described in [10]. Invasive tumours were histologically graded using the Nottingham method [46]. Tumours were then classified according to the status of the three receptors: "ER-negative/HER2-minus/PR-minus" (TNBC), "ER-/HER2 + " and "ER+/HER2-". Cases where the information on any of the three receptors was uncertain, missing or not confirmed by gene expression (ER, PR) and DNA copy number (HER2) array data were classified as "others".

### Tumour subtype assignment based on PAM50 classification

Intrinsic molecular subtype was assigned on 142 tumour samples, for which gene expression data were available. Nearest centroid classification was performed by using the *PAM50* class centroids from Parker et al. [1], using tumour gene expression data. Since the composition of our dataset was significantly enriched for triple-negative breast cancers, therefore deviating from the original data set composition used to develop PAM50 centroids, we used an iterative classification procedure based on random sampling, as described in [10].

### Genomic data processing

#### miRNA expression data

miRNA data were pre-processed using the *AgiMicroRna* (http://www.aroma-project.org) R package [47]. Quality of individual arrays was assessed by visual evaluation of RLE (relative log expression), NUSE (normalised unscaled standard error) and hierarchical clustering plots. Robust Multichip Analysis (RMA) methodology [48] was used to remove the array signal background, followed by quantile normalisation to correct for inter-arrays global differences and by miRNA level summarisation. miRNAs not detected or having saturated signal in more than 10 samples were filtered out. Replicated samples for the same patient were averaged.

#### mRNA expression data

ExonArray data pre-processing was performed on the R platform using the "aroma.affymetrix" R package (http://www.aroma-project.org). RMA was used to remove the array signal background, followed by quantile normalisation to correct for inter-arrays global differences and by gene level summarisation. For this latter step probe sets were mapped to ENSEMBL genes using the mapping file (HuEx-1_0-st-v2, U-Ensembl49, G-Affy.cdf) generated by the aroma.affymetrix team [49]. Quality of individual arrays was assessed by visual evaluation of RLE (relative log expression), NUSE (normalised unscaled standard error) and hierarchical clustering plots. Samples presenting outlier behaviour were excluded from the analyses. Replicated samples for the same patient were averaged.

#### miRNA copy-numbers

Array quality using Contrast Quality Control (CQC) and Median of the Absolute values of all Pairwise Differences (MAPD) methods provided by the Affymetrix Genotyping Console version 3.0 and low-quality arrays were excluded from further analyses. Arrays were then pre-processed using methods available in the R package "aroma.affymetrix" including techniques to remove systematic bias introduced due to PCR fragment length bias, differences in GC content and allelic cross talk. Raw DNA copy numbers were determined as shown in [50]; pre-processed signals from tumour samples were compared to the pooled data obtained from normal lymphocyte DNA from 17 patients. Raw DNA copy numbers were segmented using the circular binary segmentation method [51]. Probe-sets within copy-number aberrations that were present in more than 5% of normal samples as well as those located in common copy number polymorphisms were removed. Probe-set data were aggregated at microRNA-centric level by using the mean copy number of the probe sets within the genomic regions of the respective microRNA loci. Replicated samples for the same patient were averaged. miRNA genomic coordinates from miRBase version 18 were used (Genome assembly: GRCh37, http://mirbase.org/pub/mirbase/18/genomes/hsa.gff3). Genomic position and absolute and sample-by-sample DNA copy numbers of all miRNAs analysed are reported in Additional file 11: Table S6.

All miRNAs in the study were annotated using the largely adopted miRBase as the reference (version 18). miRNAs located on genomic transposable elements (TEs) were downloaded from a recent study of Piriyapongsa and collegues, reporting the list of miRNAs putatively and/or validated to be co-located with TEs [14]. Based on their results, we have assigned 30 miRNAs (e.g. miR-548d-5p) as co-located with TEs and therefore not assigned to a specific genomic location (Additional file 4: Table S2).

The extensive Cancer Genome Atlas Network (TCGA) work has been used as a reference for genomic regions frequently amplified/deleted as well as arm-level gains and losses (773 breast tumours), using reported data from basal-only and total breast cancers analyses [8].

miRNAs located on TEs, given their uncertain location were excluded from any analysis related to DNA copy numbers.

### Statistical analyses

A global view of the integrated analytical workflow used to generate the presented results is given in Additional file 1: Figure S1.

#### Survival analysis

Kaplan-Meier analysis was used for calculation and visualization of survival curves, and Cox-regression models followed by Wald test were used to determine the statistical association between the expression of each miRNA and distant metastases free survival (DMFS) or breast cancer specific survival (BCSS). Analyses were run separately of for different tumour groups: "all tumours", "TNBC", "ER-negative" and different Cox-regression models were used, with or without consideration of histological grade as a covariate (grouped as "low" for tumour grades 1 and 2 and "high" for grade 3). When more than one sample was available for the same patient, the average of miRNA expression values was used. Averaging of biological replicates among single measurements implied violation of the assumption of I.I.D. (independent and identically distributed) errors. However, this was done only for a very limited number of samples and comparison with results obtained from statistical analyses without using sample replicates showed no relevant differences.

To adjust for multiple testing, false discovery rate (FDR) q-values were calculated from the Wald test p-values, using Benjamin-Hochberg method. miRNA were considered to be associated to DMFS or BCSS using combined thresholds 0.002 and 0.2, for log-rank p-value and FDR q-value, respectively. To avoid any bias due to pre-surgery treatment, the 3 patients who had received neo-adjuvant treatment were excluded from the analysis. Distributions of the resulting log-rank p-values were compared with random uniform distribution (from 0 to 1), representing p-values that would be obtained by chance. With all models the obtained p-values were clearly deviating from uniform distribution with an overall bias towards low p-values, and therefore deviating from the results that would be obtained by chance (Additional file 8: Figure S5). Similar results were obtained when log-rank p-values were compared with the equivalent p-values obtained upon Monte Carlo permutation of survival data (N = 10.000). The expression data for each miRNA were scaled to unit variance when calculating proportional hazard ratios. All other covariates have been used without re-scaling. Histopathological covariates were used as follows: Histological Tumour Grading (1-2 = "low", 3 = "high"); Node positivity (yes/no); % of lymphocytic infiltration (<15% = "low", > = 15% = "high"); Tumour size (continuous variable measured in cm). With a continuous variable (e.g. miRNA expression, Tumour size), the hazard ratio indicates the change in the risk of death if the parameter in question rises by one unit (e.g. 1 cm for tumour size, 1 std for rescaled miRNA expression values). All analyses were run using R software and 'survival’ package. The two external data sets used in the analyses [17,18] were downloaded from Gene Expression Omnibus, accession IDs GSE22216 and GSE19536. The array annotation for the Illumina platform used in GSE22216 (Illumina Human v1 MicroRNA expression beadchip) was obtained from GPL8178.

#### Identification of miRNAs associated with tumour subtypes

ANOVA analysis was used to identify miRNA with differential expression across intrinsic subtypes. q-values were calculated as the ANOVA based p-values, adjusted for multiple-testing using Benjamin-Hochberg method. q-value threshold used to define association with cancer subtypes was 0.001. Similarly, Student’s t-test adjusted for multiple-testing using Benjamin-Hochberg was used to identify miRNAs differentially expressed between pairs of histopathological tumour groups (ER-negative vs ER-positive, TNBC vs non-TNBC, 1-2 tumour grade vs 3 tumour grade) (q-value threshold = 0.01).

#### Assessment of miRNA targets

For each miRNA, candidate targets were inferred using data from six different databases (*miRanda*, *PicTar*, *TargetScan*, *mirBase*, *miRTarget2*, *TarBase*[52-57]) using the *RmiR* package from R Bioconductor (http://www.bioconductor.org/).

For confirmatory evaluation of our results regarding the influence of individual miRNAs on gene sets/pathways, we used the miRTarBase, a database of manually curated miRNA-target interactions, validated experimentally by reporter assay, western blot, or microarray experiments with overexpression or knockdown of miRNA [9].

#### Association between miRNA expression and DNA copy numbers

Association between miRNA expression and perturbation in the number of DNA copies was evaluated using Spearman correlation and Wilcoxon test. The latter was used as follows: for each miRNA samples were stratified according to the DNA copy numbers in three groups: 1) copy number gains (>2.3 copies); 2) copy number losses (<1.7 copies); 3) copy number neutral. Wilcoxon rank sum test was used to assess differences in expression between samples with loss and gains compared to samples without changes. q-values were calculated as the Wilcoxon based p-values, adjusted for multiple-testing using Benjamin-Hochberg method. Thresholds used to define miRNA association with DNA copy numbers subtypes were q-value < 0.05, Spearman correlation > 0.25. The 30 miRNAs associated with TEs were excluded from the miRNA-CN correlation analysis.

#### Association between miRNA expression and gene set/pathway scores

Gene sets were extracted from public (KEGG [19], Panther [20]), commercial (GeneGO [21] and Ingenuity [22]) databases. For each gene set, a global ’signature score' was assigned to each sample for which transcriptional data were available by using the average of the gene expression level of all the genes of the signature, as described in [58]. Pathway associated with miRNAs and discussed in the paper were selected according to the following criteria: 1) absolute Spearman correlation with miRNA expression > 0.5; 2) p-value of Spearman correlation (from Spearman's *rho* statistic) less than 10-6; 3) ANOVA p-value from comparison of correlation across different groups of miRNAs (favourable vs unfavourable, PAM50 subtypes) less than 10-4. In this analysis we preferred not to adjust for multiple testing, given the strong degree of dependency between different gene sets. We used instead very conservative thresholds of associations. miRNAs and gene set scores were clustered using the hierarchical clustering algorithm, using Euclidean distance as distance metrics. The stability and statistical significance of the clusters were evaluated using the bootstrapping analysis (n = 10.000) implemented in th *pvclust* R package. Correlation values and related statistical significance between miRNA expression and gene set scores are reported in Additional file 12: Table S5. A description of all gene sets analysed and gene set scores for each sample are reported in Additional file 13: Table S7.

#### Identification of miRNAapt and assessment of targeted pathways/signatures

The workflow for the identification of miRNA exerting a control on the transcriptional levels of their targets (miRNAapt) is illustrated in Additional file 5: Figure S3. miRNAapt were selected for having an enrichment in anti-correlated candidate targets, measured as Benjamin-Hochberg corrected Fisher-test, q-value < 0.05). Anti-correlation was defined using -0.3 as the maximum threshold for Spearman correlation.

For the identification of the pathways and transcriptional signatures targeted by miRNAapt we used an adaptation of the approach described in [28], based on gene set enrichment analysis through Fisher-test. For this we used as gene sets a comprehensive compendium of published transcriptional signatures (the *Molecular Signatures Database*) [29], in addition to pathway databases used for the pathways association analysis described above. In this way a statistical link between each miRNAapt and its targeted gene sets (representing pathways or transcriptional signatures) was established. The thresholds used to assign a gene set to a miRNAapt were the following: 1) Gene set enrichment of anti-correlated targets: Fisher test p-value < 0.001 2) size of the overlap between each gene set and the list of anti-correlated miRNA targets > 3; 3) size of each gene set < 500. The procedure of identification of a miRNAapt and assignment of transcriptionally targeted gene sets was tested on 11 miRNAapt, for which the down-stream targets had been previously experimentally determined upon transfection in cultured cancer cells, followed by microarray gene expression experiments [30].

### Availability of supporting data

Patient demographics, clinical and pathological information, are reported in Additional file 3: Table S1 and include the patient’s age, survival data (DMFS, BCSS), tumour size, histological grade, nodal status, surgery and treatments and estrogen (ER), progesterone (PR) and human epidermal growth factor receptor 2 (HER2) status.

miRNA and mRNA microarray data have been deposited ins GEO public repository with ID GSE40267 (http://www.ncbi.nlm.nih.gov/geo/query/acc.cgi?acc=GSE40267). DNA copy number data and mRNA transcriptional gene set scores used for the analyses are accessible in Additional file 11: Table S6 and Additional file 13: Table S7.

## Competing interests

The authors declare that they have no competing interests.

## Authors’ contributions

Design of the study, implementation of the analytical methods, interpretation of scientific results and writing of the manuscript: EDR. Analysis of transcriptional data: BB. Samples preparation, pathological review of tumours and TMA experiments: PG and SP. Management and analysis of clinical and pathological data: AM. miRNA array experiments: ZM, GMF. Cohort selection, supervision of samples isolation, preparation and analysis: CG. Methodological supervision of Breakthrough Lab’s experimental work: PM. Analysis of SNP array data and molecular classification of tumour samples: AG. Initial conception, supervision of the study and discussion of the results: LH, DD, AT. Pathological assessment of tumour samples and critical discussion of the text: SP. Revision of the final version of the manuscript: BB, CG, AG, LH, DD, SP, AT, EDR. All authors read and approved the final manuscript.

## Supplementary Material

Additional file 1: Figure S1 Global description of the analytical workflow used to generate the presented results (in red), through the integration of genomic, clinical and pathological information. Click here for file

Additional file 2: Figure S2 Sample data and tumour classification. a) Profiling data available for different tumour classes. b) Heatmap showing different tumour characteristics: PR, ER, HER2 receptor status according to IHC; histological grade; molecular intrinsic subtype assigned using transcriptional data and the PAM50 algorithm. Click here for file

Additional file 3: Table S1 Patient and sample characteristics. These include patient treatment, age, survival data (DMFS, BCSS) and pathological features of the extracted tumour samples. Click here for file

Additional file 4: Table S2 miRNA integrated analysis results summary. *miRNA ALL* worksheet: for each miRNA represented on the Agilent chip the following information are reported: genomic position, statistical association between expression and DNA copy numbers, mean expression in different PAM50 intrinsic subtypes *miRNA assoc. with CN changes* worksheet: list of miRNA whose expression is associated with DNA copy number changes of the respective genetic loci. The occurrence of each miRNA in regions of recurrent DNA amplifications/deletions in basal-like and all-breast cancer populations, as reported in the extensive TCGA study [8], is indicated. *miRNA assoc. with PAM50 subtypes*: list of miRNA differentially expressed between different PAM50 subtypes. *PAM50 mRNAs-comparisons:* miRNA differentially expressed between different PAM50 subtypes in our and two external studies (Blenkiron et al. [24], Dvinge et al. [7]). *Guys.miR.CoxRegAllRes:* results from DMFS and BCSS survival analysis on ER-negative and TNBC samples, using different univariate and multivariate Cox-regression models (described in Additional File) (Wald test p-value, HR, and FDR corrected q-values). *ExtDataSets.miR.CoxRegAllRes:* results from DMFS survival analysis on ER-negative and TNBC samples, using different Cox-regression models in external data sets [17,18] (Wald test p-value, HR, and FDR corrected q-values). Click here for file

Additional file 5: Figure S3 Correlation between miRNAs and candidate targeted genes. Distributions of the correlations between the expression levels of individual miRNAs and their candidate target genes. Independent analyses were run using six different target prediction algorithms. Click here for file

Additional file 6: Table S3 miRNAapt and gene sets/pathways transcriptionally silenced. *miRNAapt_FDR* worksheeet: for each miRNA represented on the Agilent chip the FDR q-value of the enrichment of anti-correlated targets in the list of candidate targets obtained from different algorithms is reported. This value (MIN(q-value)<0.05) has been used to identify miRNAs exerting a transcriptional effect on their candidate targets. *miRNAapt_pathways* worksheeet: for each miRNAapt the list of gene sets (representing different pathways and transcriptional signatures) inferred to be influenced by the miRNAapt is reported. Analyses were run using different miRNA target prediction algorithm. *miRNAapt pathways miRTarBase:* for each miRNAapt the list of gene sets (representing different pathways and transcriptional signatures) inferred to be influenced by the miRNAapt is reported. Analyses were run using for each mRNAapt the list of experimentally validated targets extracted from miRTarBase. Click here for file

Additional file 7: Figure S4 Identification of miRNAapt and assessment of transcriptionally targeted pathways/signatures. a) Analytical workflow for the identification of miRNAapt and assessment of the respective transcriptionally targeted pathways and signatures b) Top: Details of step 3. Scatter plot showing the enrichment for anti-correlated candidate targets (Spearman correlation < -0.3) of all miRNAs represented on the chip. 43 miRNAapt - selected for having an FDR q-value < 0.05 - are shaded in brown. X axis: miRNAs, ordered according to increasing levels of enrichment. Y axis: -log10 of the Benjamin-Hochberg corrected Fisher-test q-value of the enrichment. Bottom: Details of step 4. Click here for file

Additional file 8 Details of the analyses carried out for associations with survival and characterization of PAM50 subtype-specific miRNAs. Click here for file

Additional file 9: Table S4 Results of univariate Cox-Regression model analyses in TNBC and ER-negative samples. Histological Tumour Grading (1-2, 3); Node positivity (pos/neg); % of lymphocytic infiltration (<15% = "low", >=15% = "high"). Tumour size (continuous variable measured in cm). With a continuous variable the hazard ratio indicates the risk change if the parameter in question rises by one unit. Click here for file

Additional file 10: Figure S18 Heat map representing the gene set enrichments (expressed as the -log10 of the Fisher-test p-value) of experimentally validated targeted gene sets (rows), targeted by different miRNAapt (columns) through experimental analyses (as reported in [29]). Each gene set represents the list of genes down-regulated upon over-expression of a miRNA in the HCT116 cell line, as inferred from microarray experiments after 10 or 24 hours from transfection. The analysis shows that the sets of genes determined to be targeted by each miRNA are also inferred to be directly targeted - with high statistical significance - upon the application of our method to the same miRNA. Links between miRNAapt and targeted sets are highlighted using corresponding colours. Click here for file

Additional file 11: Table S6 Genomic position and absolute and sample-by-sample DNA copy numbers of all miRNA represented on the Agilent chip. DNA copy numbers were obtained through the processing of SNP Array data (see Methods). Click here for file

Additional file 12: Table S5 Spearman values and related statistical significance of the correlation between miRNA expression and gene set scores. Only correlations with absolute values > 0.4 and p-values < 10-4 are reported. Click here for file

Additional file 13: Table S7 *GENE_SETS DESCRIPTION* worksheet: description of all gene sets used in the analyses. *GENE_SETS_SCORES* worksheet: sample-by-sample scores of all gene sets used in the analyses. Click here for file
