# Supplementary material for: Integrated genomic analysis of triple-negative breast cancers reveals novel microRNAs associated with clinical and molecular phenotypes and sheds light on the pathways they control
Source: BMC Genomics. 2013 Sep 23;14:643. doi: 10.1186/1471-2164-14-643 (PMC4008358; doi:10.1186/1471-2164-14-643)
Supplement: Additional file 1: Figure S1 — Global description of the analytical workflow used to generate the presented results (in red), through the integration of genomic, clinical and pathological information. [file 1471-2164-14-643-S1.pptx]

## Slide 1
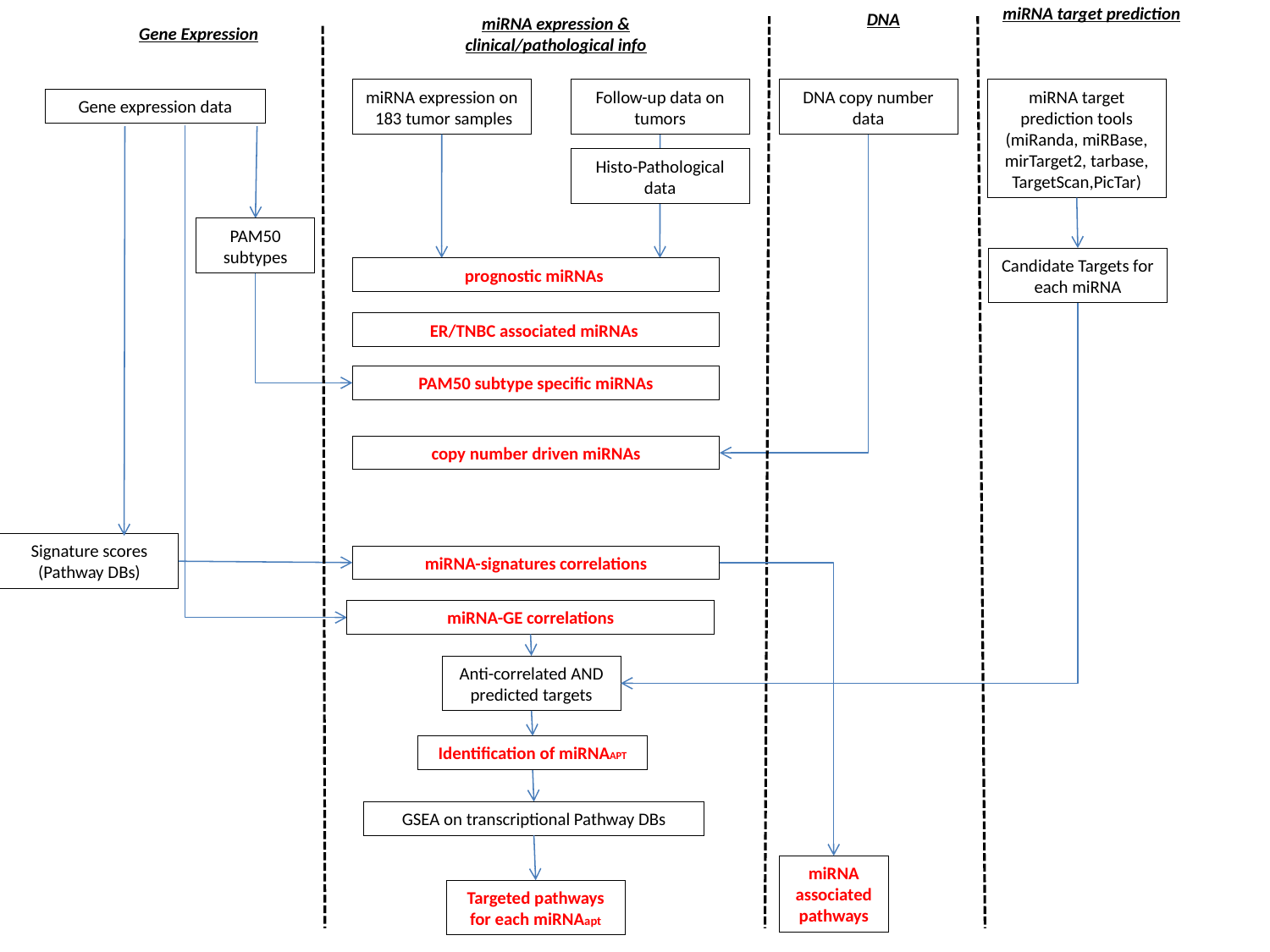

miRNA target prediction
DNA
miRNA expression & clinical/pathological info
Gene Expression
miRNA expression on 183 tumor samples
Follow-up data on tumors
DNA copy number data
miRNA target prediction tools (miRanda, miRBase, mirTarget2, tarbase, TargetScan,PicTar)
Gene expression data
Histo-Pathological data
PAM50 subtypes
Candidate Targets for each miRNA
prognostic miRNAs
ER/TNBC associated miRNAs
PAM50 subtype specific miRNAs
copy number driven miRNAs
Signature scores (Pathway DBs)
miRNA-signatures correlations
miRNA-GE correlations
Anti-correlated AND predicted targets
Identification of miRNAAPT
GSEA on transcriptional Pathway DBs
miRNA associated pathways
Targeted pathways for each miRNAapt
