# Supplementary material for: Integrated genomic analysis of triple-negative breast cancers reveals novel microRNAs associated with clinical and molecular phenotypes and sheds light on the pathways they control
Source: BMC Genomics. 2013 Sep 23;14:643. doi: 10.1186/1471-2164-14-643 (PMC4008358; doi:10.1186/1471-2164-14-643)
Supplement: Additional file 2: Figure S2 — Sample data and tumour classification. a) Profiling data available for different tumour classes. b) Heatmap showing different tumour characteristics: PR, ER, HER2 receptor status according to IHC; histological grade; molecular intrinsic subtype assigned using transcriptional data and the PAM50 algorithm. [file 1471-2164-14-643-S2.pptx]

## Slide 1
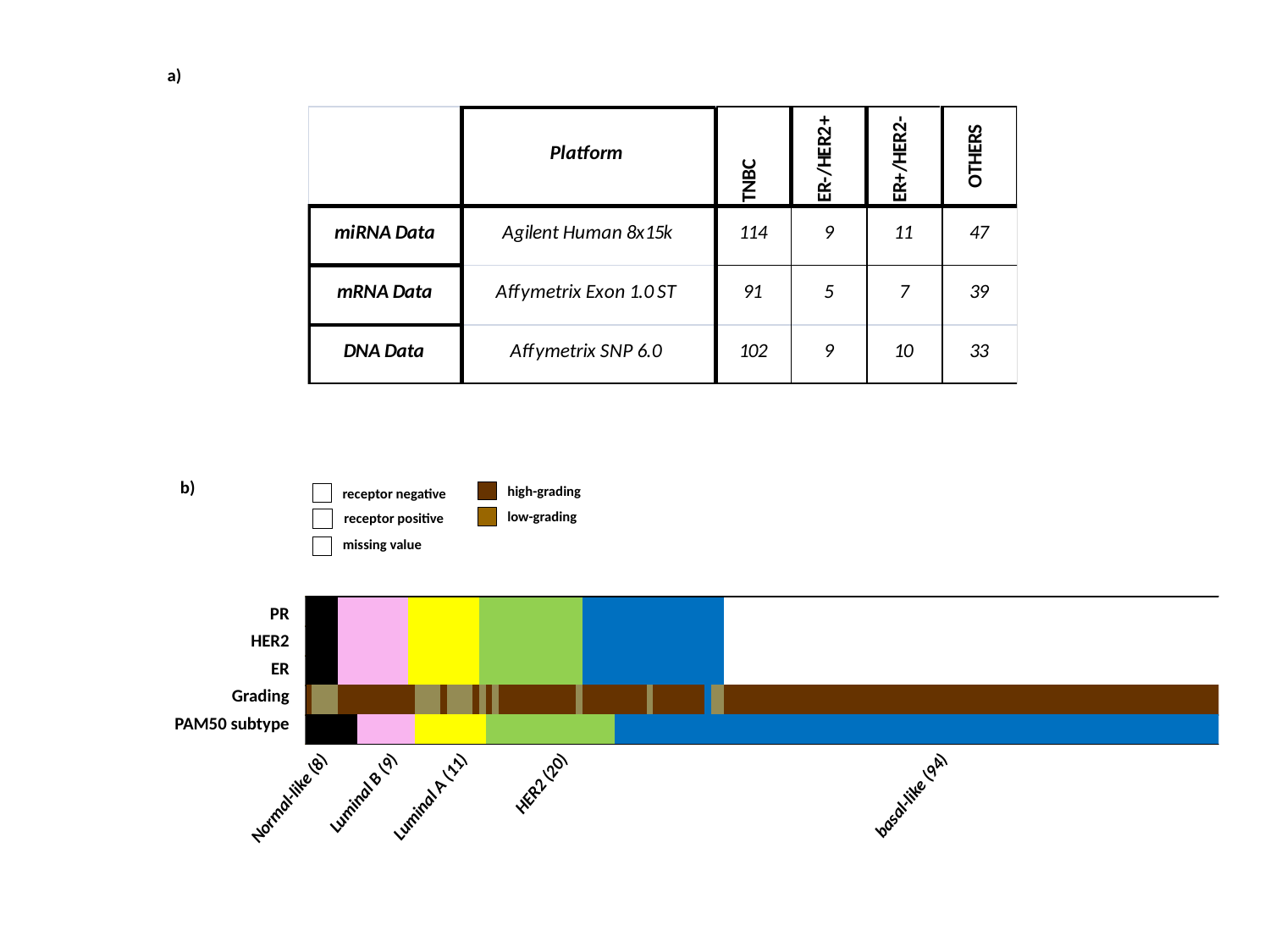

a)
b)
high-grading
receptor negative
low-grading
receptor positive
missing value
PR
HER2
ER
Grading
PAM50 subtype
HER2 (20)
Luminal A (11)
Normal-like (8)
Luminal B (9)
basal-like (94)
