# Supplementary figures and images for: Integrated genomic analysis of triple-negative breast cancers reveals novel microRNAs associated with clinical and molecular phenotypes and sheds light on the pathways they control
Source: BMC Genomics. 2013 Sep 23;14:643. doi: 10.1186/1471-2164-14-643 (PMC4008358; doi:10.1186/1471-2164-14-643)

**miranda**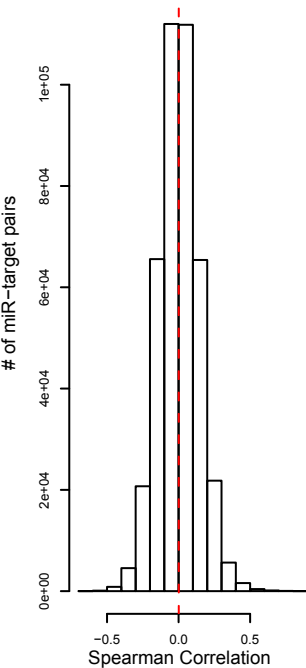**mirbase**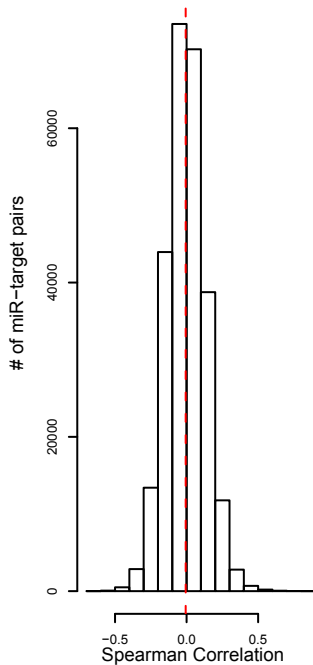**mirtarget2**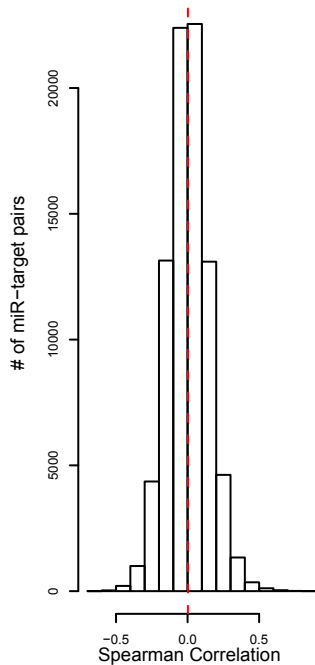**pictar**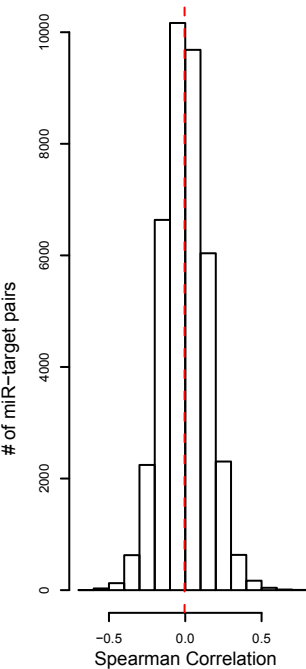**tarbase**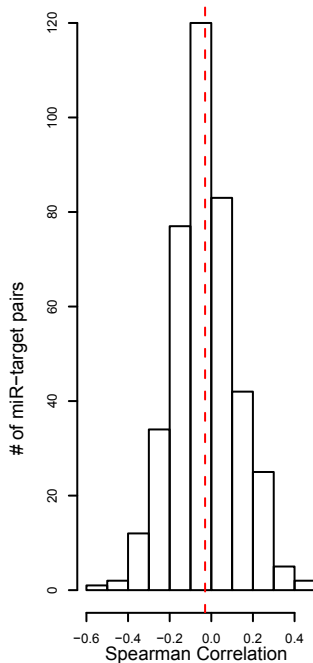**targetscan**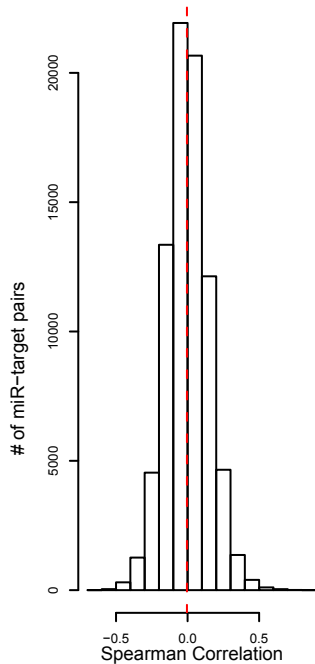

Supplement: Additional file 5: Figure S3 — Correlation between miRNAs and candidate targeted genes. Distributions of the correlations between the expression levels of individual miRNAs and their candidate target genes. Independent analyses were run using six different target prediction algorithms. [file 1471-2164-14-643-S5.pdf]

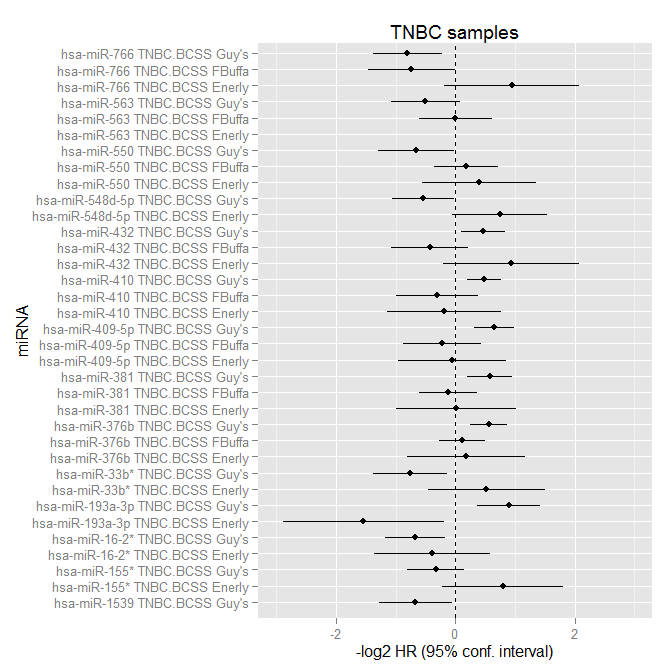

Supplement: Additional file 8 — Details of the analyses carried out for associations with survival and characterization of PAM50 subtype-specific miRNAs. [file 1471-2164-14-643-S8.zip › 4069309791507884_add8/4069309791507884_figS10.tiff]

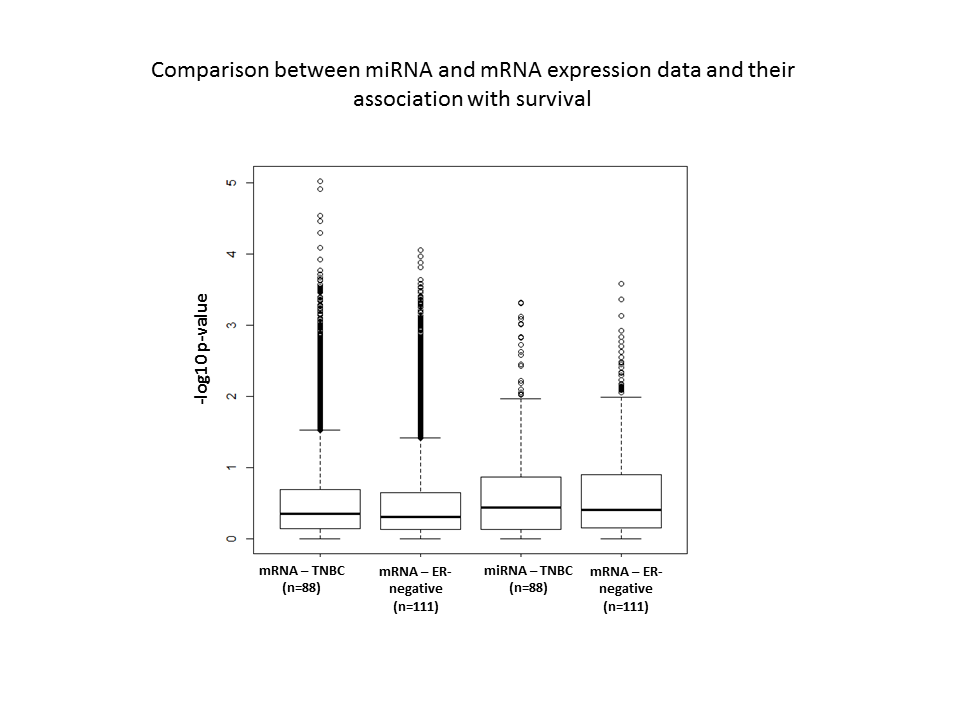

Supplement: Additional file 8 — Details of the analyses carried out for associations with survival and characterization of PAM50 subtype-specific miRNAs. [file 1471-2164-14-643-S8.zip › 4069309791507884_add8/4069309791507884_figS11.tiff]

## Slide 1
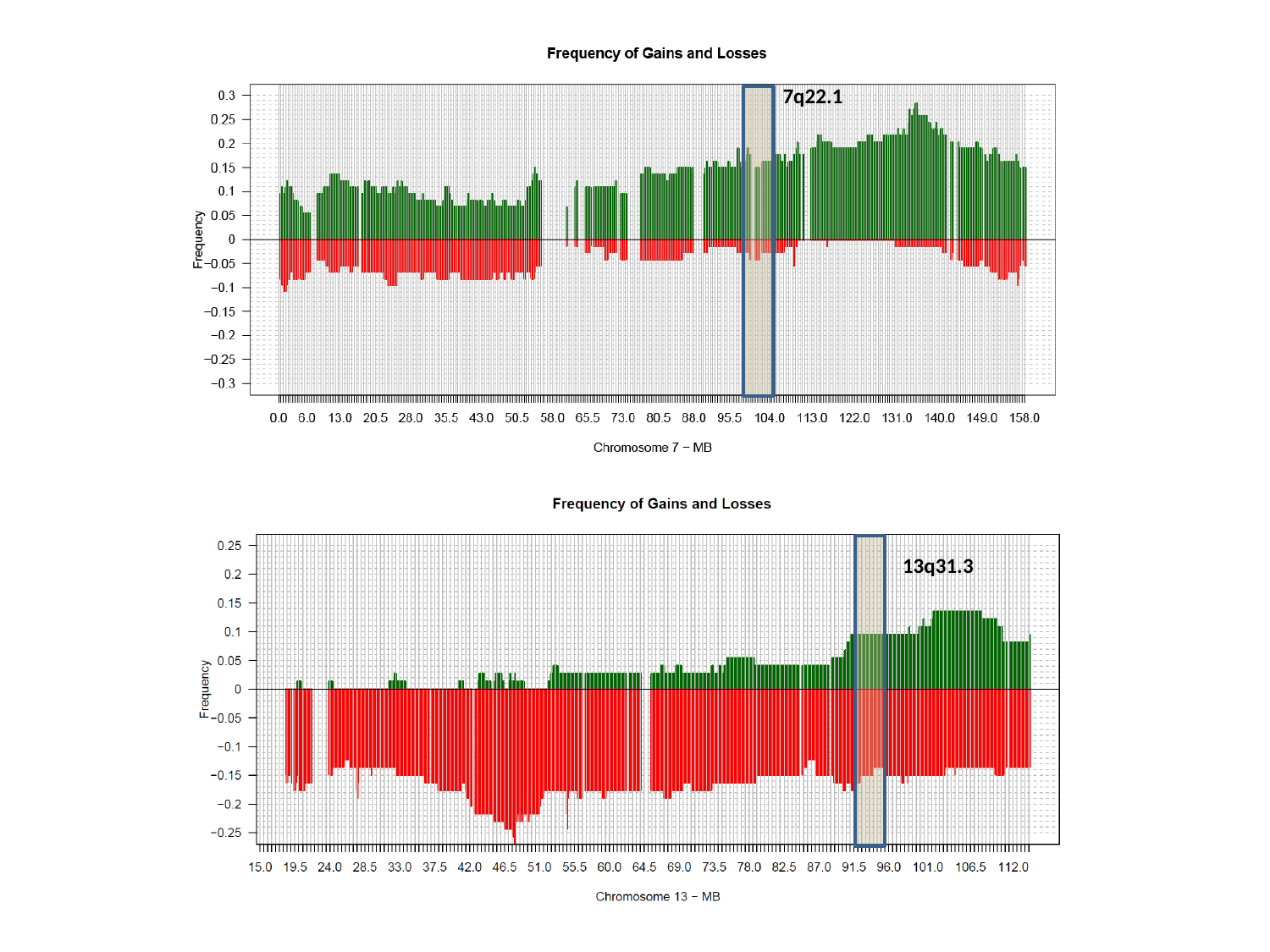

7q22.1
 13q31.3

Supplement: Additional file 8 — Details of the analyses carried out for associations with survival and characterization of PAM50 subtype-specific miRNAs. [file 1471-2164-14-643-S8.zip › 4069309791507884_add8/4069309791507884_figS14.pptx]

## Slide 1
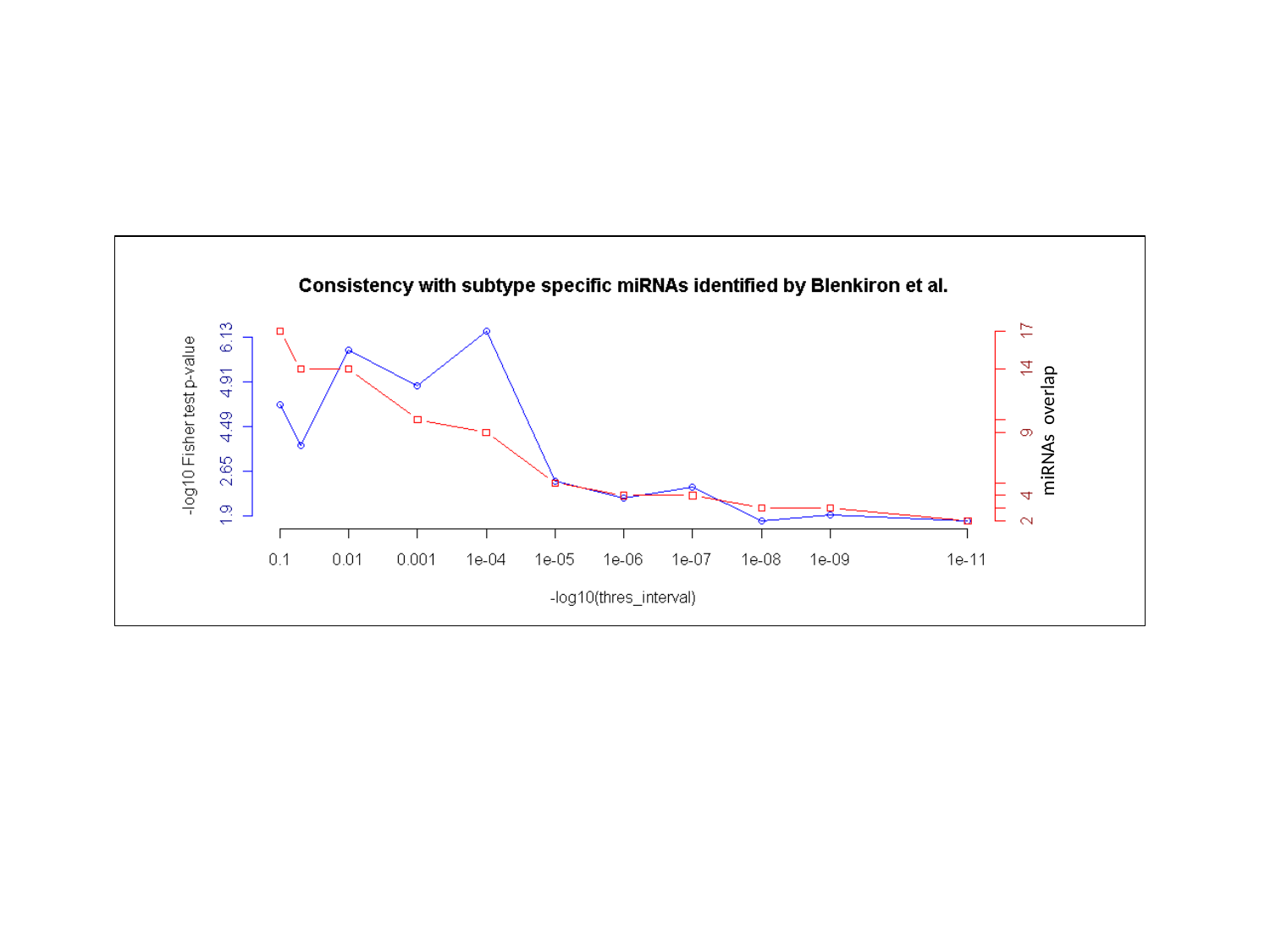

miRNAs overlap

Supplement: Additional file 8 — Details of the analyses carried out for associations with survival and characterization of PAM50 subtype-specific miRNAs. [file 1471-2164-14-643-S8.zip › 4069309791507884_add8/4069309791507884_figS16.pptx]

## Slide 1
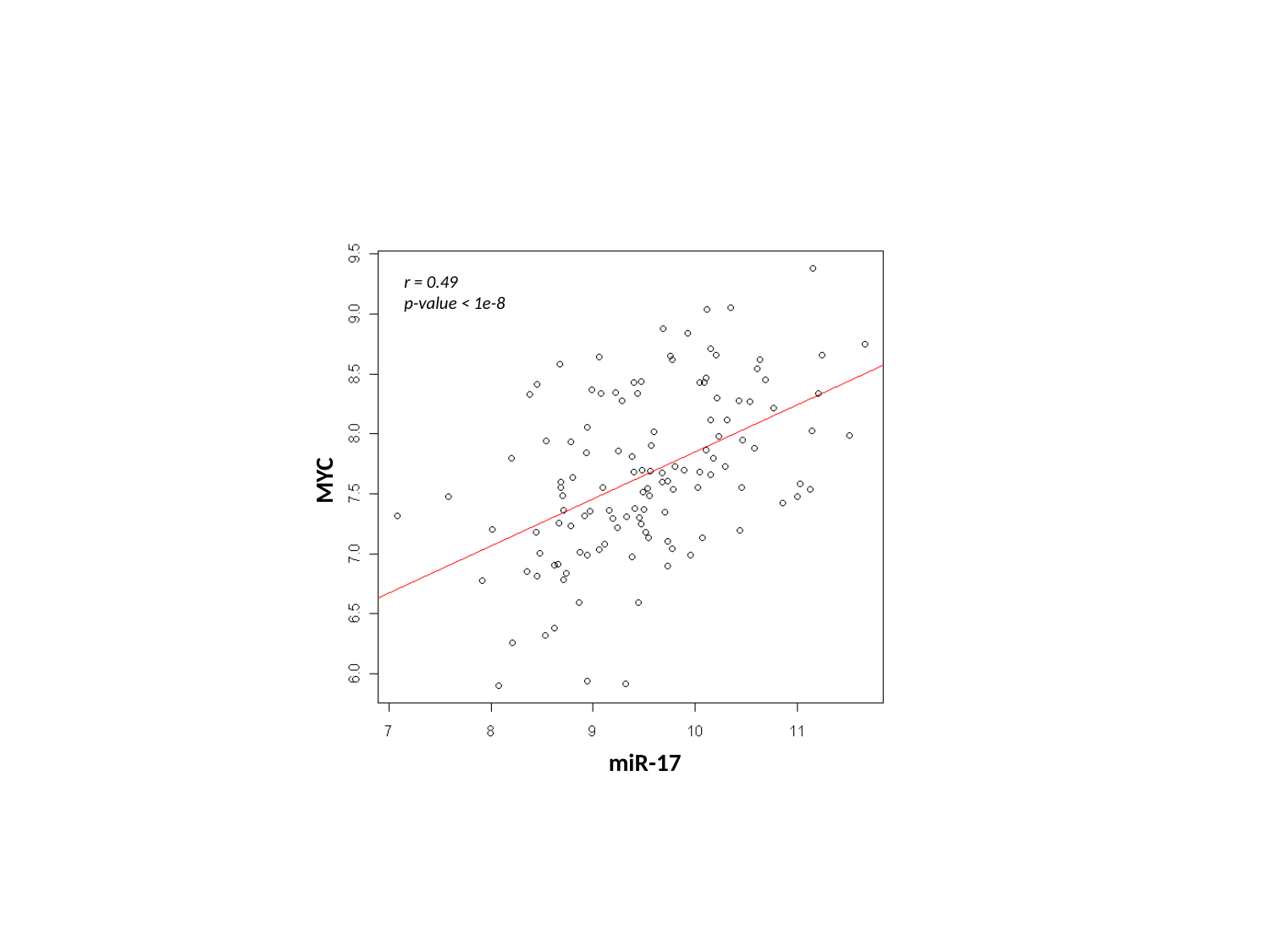

r = 0.49
p-value < 1e-8
MYC
miR-17

Supplement: Additional file 8 — Details of the analyses carried out for associations with survival and characterization of PAM50 subtype-specific miRNAs. [file 1471-2164-14-643-S8.zip › 4069309791507884_add8/4069309791507884_figS17.pptx]

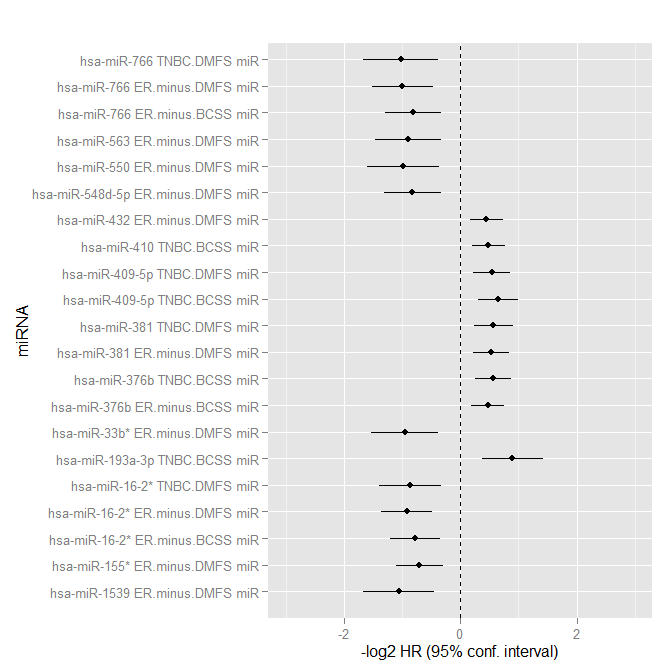

Supplement: Additional file 8 — Details of the analyses carried out for associations with survival and characterization of PAM50 subtype-specific miRNAs. [file 1471-2164-14-643-S8.zip › 4069309791507884_add8/4069309791507884_figS5.tiff]

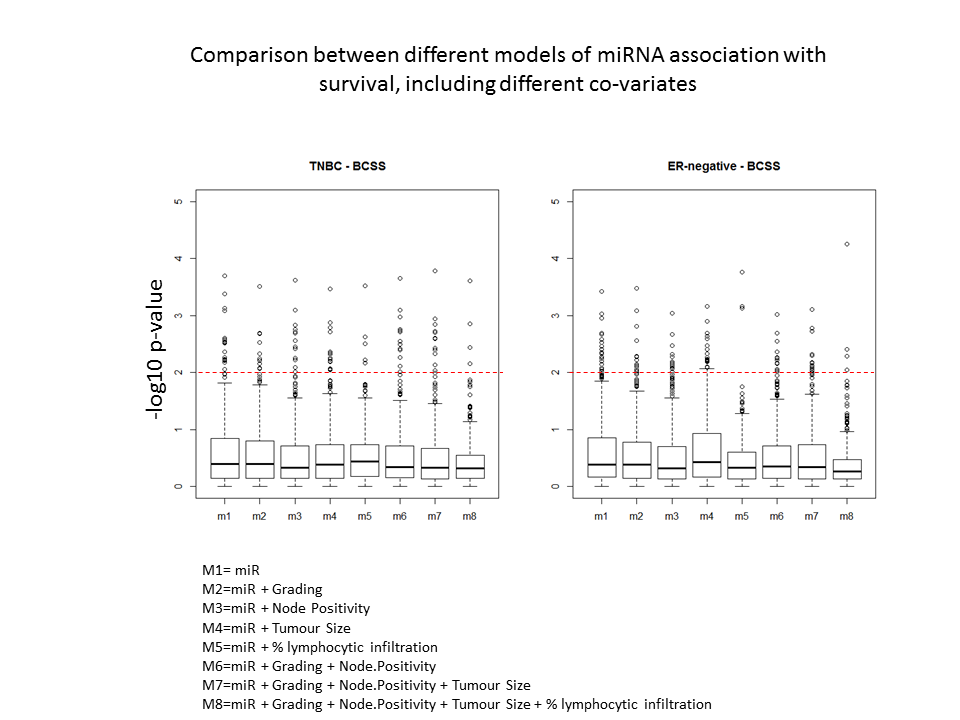

Supplement: Additional file 8 — Details of the analyses carried out for associations with survival and characterization of PAM50 subtype-specific miRNAs. [file 1471-2164-14-643-S8.zip › 4069309791507884_add8/4069309791507884_figS7.tiff]

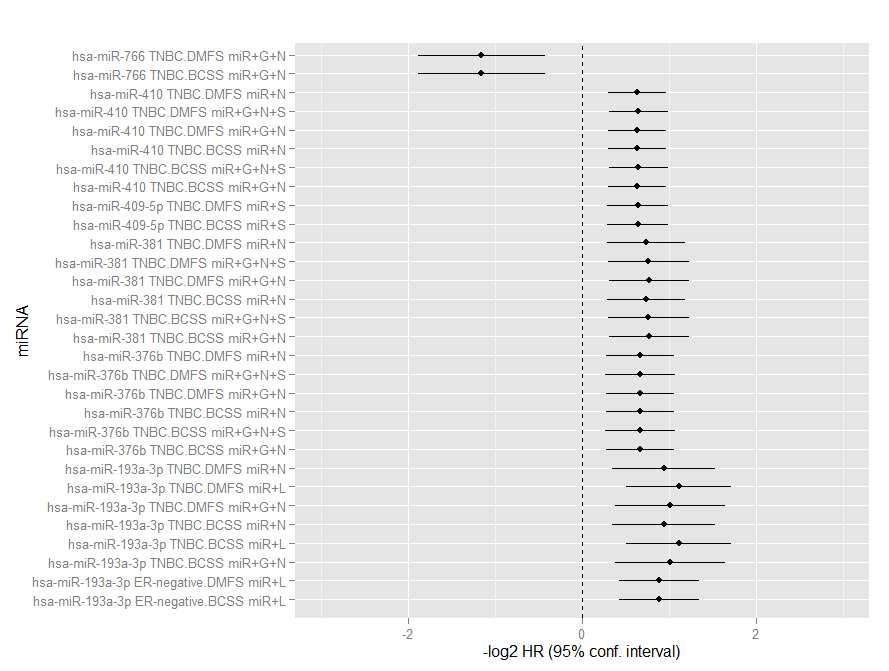

Supplement: Additional file 8 — Details of the analyses carried out for associations with survival and characterization of PAM50 subtype-specific miRNAs. [file 1471-2164-14-643-S8.zip › 4069309791507884_add8/4069309791507884_figS8.tiff]

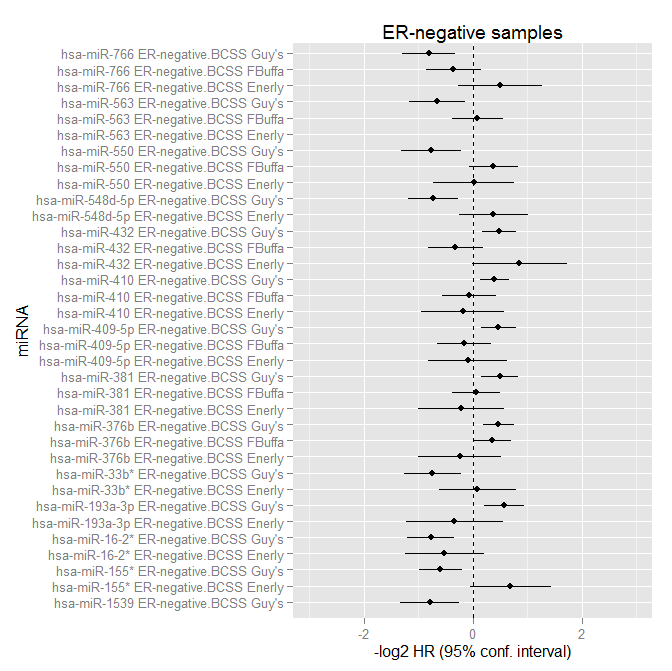

Supplement: Additional file 8 — Details of the analyses carried out for associations with survival and characterization of PAM50 subtype-specific miRNAs. [file 1471-2164-14-643-S8.zip › 4069309791507884_add8/4069309791507884_figS9.tiff]

*p-val*

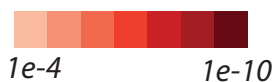

Basal-like

Luminal

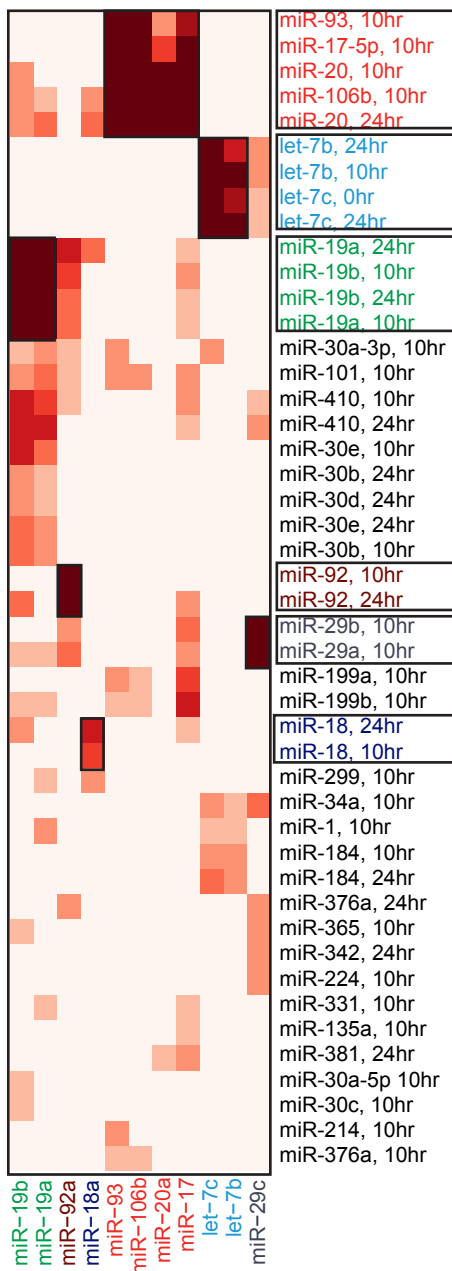

Supplement: Additional file 10: Figure S18 — Heat map representing the gene set enrichments (expressed as the -log10 of the Fisher-test p-value) of experimentally validated targeted gene sets (rows), targeted by different miRNAapt (columns) through experimental analyses (as reported in [29]). Each gene set represents the list of genes down-regulated upon over-expression of a miRNA in the HCT116 cell line, as inferred from microarray experiments after 10 or 24 hours from transfection. The analysis shows that the sets of genes determined to be targeted by each miRNA are also inferred to be directly targeted - with high statistical significance - upon the application of our method to the same miRNA. Links between miRNAapt and targeted sets are highlighted using corresponding colours. [file 1471-2164-14-643-S10.pdf]
