# Supplementary material for: Integrated genomic analysis of triple-negative breast cancers reveals novel microRNAs associated with clinical and molecular phenotypes and sheds light on the pathways they control
Source: BMC Genomics. 2013 Sep 23;14:643. doi: 10.1186/1471-2164-14-643 (PMC4008358; doi:10.1186/1471-2164-14-643)
Supplement: Additional file 7: Figure S4 — Identification of miRNAapt and assessment of transcriptionally targeted pathways/signatures. a) Analytical workflow for the identification of miRNAapt and assessment of the respective transcriptionally targeted pathways and signatures b) Top: Details of step 3. Scatter plot showing the enrichment for anti-correlated candidate targets (Spearman correlation < -0.3) of all miRNAs represented on the chip. 43 miRNAapt - selected for having an FDR q-value < 0.05 - are shaded in brown. X axis: miRNAs, ordered according to increasing levels of enrichment. Y axis: -log10 of the Benjamin-Hochberg corrected Fisher-test q-value of the enrichment. Bottom: Details of step 4. [file 1471-2164-14-643-S7.pptx]

## Slide 1
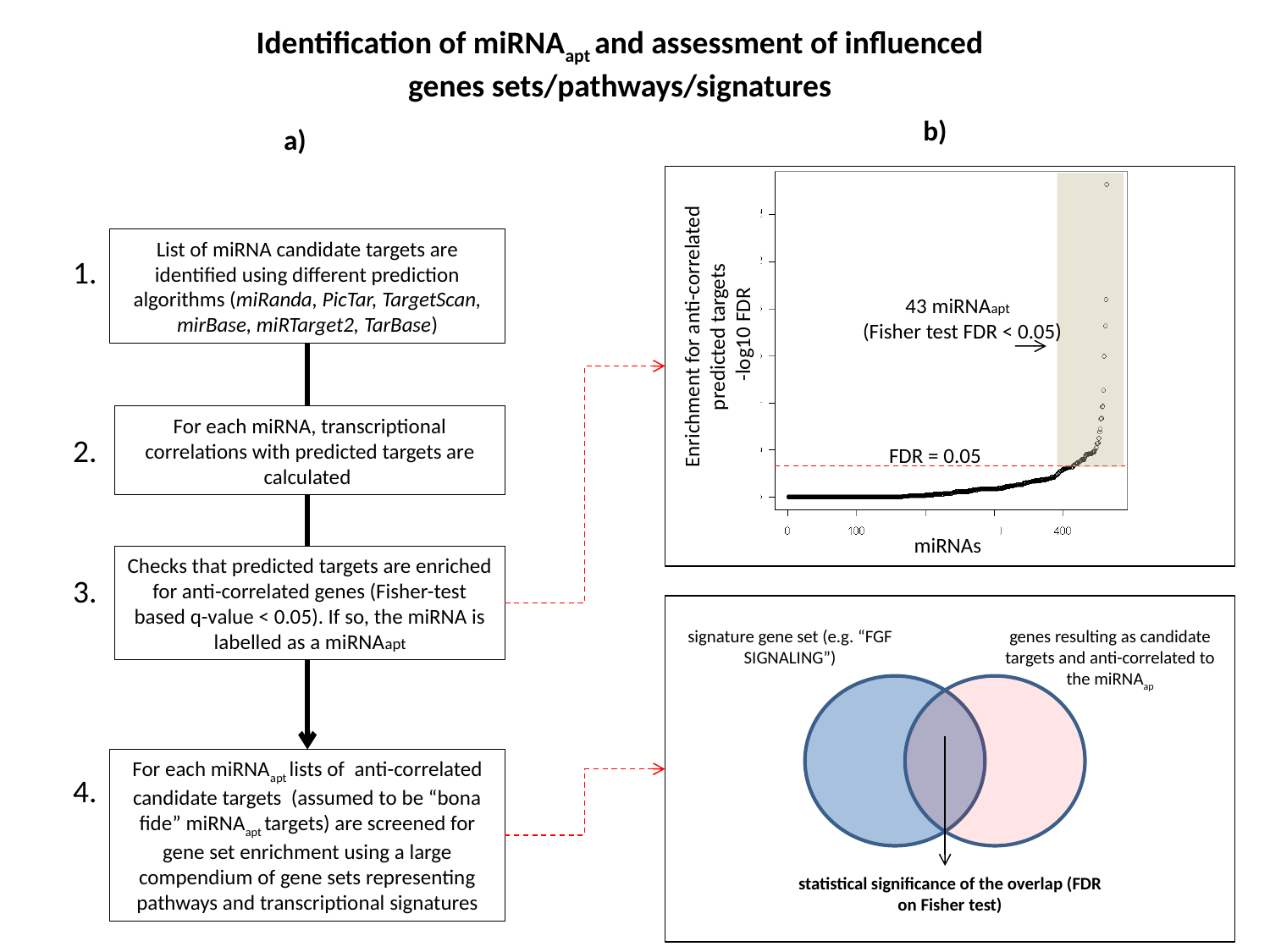

Identification of miRNAapt and assessment of influenced genes sets/pathways/signatures
b)
a)
List of miRNA candidate targets are identified using different prediction algorithms (miRanda, PicTar, TargetScan, mirBase, miRTarget2, TarBase)
1.
43 miRNAapt
 (Fisher test FDR < 0.05)
Enrichment for anti-correlated predicted targets
 -log10 FDR
For each miRNA, transcriptional correlations with predicted targets are calculated
2.
FDR = 0.05
miRNAs
Checks that predicted targets are enriched for anti-correlated genes (Fisher-test based q-value < 0.05). If so, the miRNA is labelled as a miRNAapt
3.
signature gene set (e.g. “FGF SIGNALING”)
genes resulting as candidate targets and anti-correlated to the miRNAap
For each miRNAapt lists of anti-correlated candidate targets (assumed to be “bona fide” miRNAapt targets) are screened for gene set enrichment using a large compendium of gene sets representing pathways and transcriptional signatures
4.
statistical significance of the overlap (FDR on Fisher test)
