# Supplementary material for: Integrated genomic analysis of triple-negative breast cancers reveals novel microRNAs associated with clinical and molecular phenotypes and sheds light on the pathways they control
Source: BMC Genomics. 2013 Sep 23;14:643. doi: 10.1186/1471-2164-14-643 (PMC4008358; doi:10.1186/1471-2164-14-643)
Supplement: Additional file 8 — Details of the analyses carried out for associations with survival and characterization of PAM50 subtype-specific miRNAs. [file 1471-2164-14-643-S8.zip › 4069309791507884_add8/4069309791507884_add26.docx]

**APPENDIX FILE 1**

**SURVIVAL ANALYSES**

1. Survival analysis using miRNA expression data

Cox-regression univariate analysis was carried out to identify miRNAs whose expression was associated with clinical outcome. Analyses were run separately for the ER-negative and its triple-negative sub-cohorts, with respect to breast cancer specific survival (BCSS) and distant metastases-free survival (DMFS) clinical end points.

As result, 14 miRNAs associated with prognosis were identified in ER-negative tumours; of these 7 were also significant in the sub-set of triple-negative tumours (Figs. 2-3 and S5). Analysis of the resulting p-value distributions - all skewed towards low p-values - indicated that the selection of prognostic miRNA was not a result of the high dimensional nature of the data (Fig. S6).

1. Survival analysis using only histopathological information

Survival analysis has been run including all pathological information available. The following covariates were evaluated in a Cox Regression additive model, assuming no interactions:

- Histological Tumour Grading
- Node positivity
- Tumour size
- % of lymphocytic infiltration

Two independent analyses were run on ER-negative and the sub-cohort of TNBC samples (Table S4).

*Results in ER-negative samples:*

- Node positivity alone is a negative prognostic factor.
- Tumor size alone is a negative prognostic factor.
- % of lymphocytic infiltration alone is a positive prognostic factor (higher values associated with better prognosis).

*Results in TNBC samples:*

- In the sub-cohort of TNBC samples, association between node positivity and % of lymphocytic infiltration with prognosis could also be demonstrated

1. Analysis using combined histopathological and miRNA expression data

Survival analysis has been run including individual miRNA expression and all pathological information available. miRNA expression and the following covariates were evaluated in a Cox Regression analysis (additive model, assuming no interactions):

- Histological Tumour Grading
- Node positivity
- Tumour size
- % of lymphocytic infiltration

We compared the results obtained from Cox-regression models when miRNAs were used on their own, or in association with individual or multiple co-variates. Boxplots of p-values derived from different models were then compared (Fig. S6) showing a clear impact on the percentage of lymphocytic infiltration on the association of miRNAs with prognosis (models M5 and M8, Fig. S7). In other words, miRNAs showed to be less informative with respect to prognosis when evaluated in association with lymphocytic infiltration information.

On evaluation of the additive models, among the 14 miRNAs collectively identified to be associated with prognosis alone, 5 retained their prognostic value when evaluated together with grading, node positivity and tumour size (miR-376b, miR-381, miR-409-5p, miR-410, and miR-766) and only 1 (miR-193a-3p) when lymphocytic infiltration was also considered, either in TNBC or in the wider group of ER-negative samples (including TNBC samples) or both (Fig. S8, Table S2).

1. Comparative assessment of the 14 prognostic miRNAs identified in our data set, using 2 external miRNA data sets.

We have analysed two independent miRNA data sets: one from Buffa et al., including 82 ER-negative, of which 37 are triple-negative [3]; the second from Enerly et al,, including 32 ER-negative sample, of which 21 are triple-negative [4]. All analyses were run independently in ER-negative and triple-negative subsets. For each of the 14 miRNA we identified to be associated with prognosis in our cohort, we therefore checked their behaviour in the other two data sets. Results showed broad lack of reproducibility between the 3 sample cohorts (Figs. S9-S10), confirming the elusive nature of miRNAs when endeavours are made to validate their prognostic associations in external studies, as recently resulting also from the study of Dvinge et al. [[1](#_ENREF_1)].

1. Assessment of how the best prognosis associated transcripts compare to the identified miRNAs in a Cox regression analysis to be associated to clinical outcome

The comparison between these two types of information with regard to their association with clinical outcome is not straightforward, given the higher number of mRNA data compared to miRNA’s. After having re-shaped to 2 data sets to impose the same sample size in both (ER-negative (n=111), TNBC (n=88) we run Cox-regression survival analysis on TNBC and ER samples using gene expression and miRNA data separately, without including pathological information in the model. By using FDR for multiple testing correction (which does not take into account the internal dependencies) miRNAs and mRNA expression values showed similar performance (FDR ~ 0.1in both cases). When uncorrected p-values were evaluated, mRNA outperformed miRNAs with p-val < 10^-5 against p-val<10^-3.

When we compared the distributions of p-values derived from miRNA and mRNA analyses, showing no significant differences between the two (Figure S11).

**ANALYSIS OF SUBTYPE SPECIFIC miRNAs**

In order to get a global view of the behaviour of sub-type specific miRNAs, we computed a comparative assessment of their expression in ER-positive/ER-negative, triple-negative/non-triple-negative tumours, high/low-grade tumours (Fig. S12). As expected from the relationships existing between these different classification schemes and pathological tumour features, miRNAs associated with the basal-like intrinsic subtype tend to be also over-expressed in triple-negative and high-grade tumours.

*miR-17-92 and miR-106b-25 clusters*

Notably, among the miRNAs most up-regulated in basal-like tumours we observed members of the miR-17-92 cluster (miR-20a, miR-92a, miR-17/*, miR-19a/b, miR-18a) and its paralog miR-106b-25 (miR-106b, miR-93). These two miRNA clusters have been reported to be amplified and/or over-expressed in a variety of hematopoietic and solid tumours and are emerging as key modulators of various cancer –associated processes, including proliferation, apoptosis and angiogenesis (reviewed in [[2](#_ENREF_2), [3](#_ENREF_3)]). The human miR-17-92 cluster is located in the third intron of a primary transcript known as C13orf25 [[4](#_ENREF_4)] at genomic location 13q31.3, while miR-106b-25 cluster is located within the 13th intron of the protein-coding gene MCM7, at 7q22.1 (Fig. S13a).

miRNA-mRNA data analysis showed very high correlations between these miRNA clusters and their respective host transcripts (Fig. S13b), indicating these miRNAs to share the promoter of their host genes. This observation is not obvious as several lines of evidence point towards independent expression of many intronic miRNAs from their host genes and show that intronic microRNAs may have their own promoters [[5](#_ENREF_5), [6](#_ENREF_6)].

We also detected DNA copy number gains of these regions in the basal-like subtype (Fig. S14), associated with miRNA over-expression (Fig. S15).

**Validation of subtype specific miRNA in an external data set**

Our results on subtype specific miRNAs were compared with those obtained by Blenkiron et al. on a data set of 93 breast tumours [[7](#_ENREF_7)]. By using FDR threshold=0.01 in our analysis, an overlap of 14 miRNAs (out of the 23 identified by Blenkiron) was obtained (Fisher test p-val < 10-6). The statistical significance of the overlap is maintained when different thresholds to select subtype-specific miRNAs from our study are used (Fig. S16). These data demonstrate an overall close convergence of our results with those obtained from the Blenkiron’s independent data set. Similar comparative assessment was run using the recent results reported in [[1](#_ENREF_1)]. As a result, 41 out of the 88 subtype-specific miRNAs they have found overlap with our study (Fisher test p-val < 10-8) (Table S2).

**Functional experiments on miRNAs identified as basal-like specific**

Among many gene sets we found to be influenced by miR-17-92 and miR-106b-25 clusters, are cancer-related gene sets mirroring the activation levels of MYC, TGF-β, mTOR, PTEN and the AKT pathways. The interference of miR-17-92 and miR-106b-25 with the TGF-β pathway was supported by studies run on different cancer settings, including gastric cancer [[8](#_ENREF_8)], mesenchimal [[9](#_ENREF_9)] and neuroblastoma [[3](#_ENREF_3), [10](#_ENREF_10)]. In neuroblastoma cells the effect of over-expression of individual miR-17-92 miRs have been assessed, by examining the list of down-regulated proteins through gene set enrichment analysis [[10](#_ENREF_10)]. Following an independent route, the authors reached our same conclusions on the interference of the miR-17-92 cluster with the TGF-β signalling pathway. Also in agreement with our results, their analysis showed the interference of miR-17 with the ESR1 pathway.

The relationship between miR-17-92, MYC, PTEN and mTOR pathway has also been largely documented [[3](#_ENREF_3)]. Experimental evidences showed that the miR-17-92 cluster exerts control on cell proliferation through the activation of the MYC and mTOR pathways and the co-ordinated silencing of the PTEN tumour suppressor [[11](#_ENREF_11)].Using a mouse model of colon cancer, Dews et al. demonstrated that the angiogenic activity of c-Myc is partially mediated by downstream activation of the miR-17-92 cluster [[12](#_ENREF_12)]. A model was proposed describing a feedback loop whereby MYC induces the transcription of miR-17-92 and is inhibited at the post-transcriptional level by the same miRNA cluster members [[13](#_ENREF_13)]. Our data support the transcriptional induction of MYC on the miR-17-92 cluster (Fig. S17) and suggest that miR-17-92 miRNAs might control many of the genes targeted by MYC, adding an additional layer of complexity to the proposed feedback loop model.

Olive et al. showed also that a in mouse model B-cell lymphomas, the PTEN pathway is down-regulated in response to elevated levels of miR-19, from the miR-17-92 cluster [[11](#_ENREF_11)]. As for the interference between miR-93 and elements of the AKT pathway we detected (such as CDKN1A and JAK1), supporting results can be found in the recent work of Fu et al, in ovarian cells [[14](#_ENREF_14)]. Here the authors demonstrated the direct interfering effect of miR-93 on the PTEN/AKT pathway using over-expression/knock-down experiments.

Our analyses also indicated the targeting of an epithelial-mesenchymal transition (EMT) signature by miR-17, miR-19a/b and miR-106b. External evidences report this signature to be up-regulated specifically in metaplastic carcinomas, but not in other basal-like tumours [[15](#_ENREF_15)]. Taken together these observations suggest a role of these miRNAs in lowering the transcriptional levels of EMT genes in basal-like tumours. We found miR-19b to target elements of focal adhesion and endothelium while miR-92a was found to be involved in the regulation of the cytoskeleton, consistent with the reported role of miR-17-92 in the remodelling of the extracellular matrix and the sustaining of angiogenesis [[16](#_ENREF_16)].

1. Dvinge H, Git A, Graf S, Salmon-Divon M, Curtis C, Sottoriva A, Zhao Y, Hirst M, Armisen J, Miska EA *et al*: **The shaping and functional consequences of the microRNA landscape in breast cancer**. *Nature* 2013, **497**(7449):378-382.

2. Olive V, Jiang I, He L: **mir-17-92, a cluster of miRNAs in the midst of the cancer network**. *The international journal of biochemistry & cell biology* 2010, **42**(8):1348-1354.

3. Mendell JT: **miRiad roles for the miR-17-92 cluster in development and disease**. *Cell* 2008, **133**(2):217-222.

4. Ota A, Tagawa H, Karnan S, Tsuzuki S, Karpas A, Kira S, Yoshida Y, Seto M: **Identification and characterization of a novel gene, C13orf25, as a target for 13q31-q32 amplification in malignant lymphoma**. *Cancer Res* 2004, **64**(9):3087-3095.

5. Ozsolak F, Poling LL, Wang Z, Liu H, Liu XS, Roeder RG, Zhang X, Song JS, Fisher DE: **Chromatin structure analyses identify miRNA promoters**. *Genes Dev* 2008, **22**(22):3172-3183.

6. Corcoran DL, Pandit KV, Gordon B, Bhattacharjee A, Kaminski N, Benos PV: **Features of mammalian microRNA promoters emerge from polymerase II chromatin immunoprecipitation data**. *PLoS One* 2009, **4**(4):e5279.

7. Blenkiron C, Goldstein LD, Thorne NP, Spiteri I, Chin SF, Dunning MJ, Barbosa-Morais NL, Teschendorff AE, Green AR, Ellis IO *et al*: **MicroRNA expression profiling of human breast cancer identifies new markers of tumor subtype**. *Genome Biol* 2007, **8**(10):R214.

8. Petrocca F, Vecchione A, Croce CM: **Emerging role of miR-106b-25/miR-17-92 clusters in the control of transforming growth factor beta signaling**. *Cancer Res* 2008, **68**(20):8191-8194.

9. Li L, Shi JY, Zhu GQ, Shi B: **MiR-17-92 cluster regulates cell proliferation and collagen synthesis by targeting TGFB pathway in mouse palatal mesenchymal cells**. *J Cell Biochem* 2012, **113**(4):1235-1244.

10. Mestdagh P, Bostrom AK, Impens F, Fredlund E, Van Peer G, De Antonellis P, von Stedingk K, Ghesquiere B, Schulte S, Dews M *et al*: **The miR-17-92 microRNA cluster regulates multiple components of the TGF-beta pathway in neuroblastoma**. *Mol Cell* 2010, **40**(5):762-773.

11. Olive V, Bennett MJ, Walker JC, Ma C, Jiang I, Cordon-Cardo C, Li QJ, Lowe SW, Hannon GJ, He L: **miR-19 is a key oncogenic component of mir-17-92**. *Genes Dev* 2009, **23**(24):2839-2849.

12. Dews M, Homayouni A, Yu D, Murphy D, Sevignani C, Wentzel E, Furth EE, Lee WM, Enders GH, Mendell JT *et al*: **Augmentation of tumor angiogenesis by a Myc-activated microRNA cluster**. *Nat Genet* 2006, **38**(9):1060-1065.

13. Aguda BD, Kim Y, Piper-Hunter MG, Friedman A, Marsh CB: **MicroRNA regulation of a cancer network: consequences of the feedback loops involving miR-17-92, E2F, and Myc**. *Proc Natl Acad Sci U S A* 2008, **105**(50):19678-19683.

14. Fu X, Tian J, Zhang L, Chen Y, Hao Q: **Involvement of microRNA-93, a new regulator of PTEN/Akt signaling pathway, in regulation of chemotherapeutic drug cisplatin chemosensitivity in ovarian cancer cells**. *FEBS letters* 2012, **586**(9):1279-1286.

15. Taube JH, Herschkowitz JI, Komurov K, Zhou AY, Gupta S, Yang J, Hartwell K, Onder TT, Gupta PB, Evans KW *et al*: **Core epithelial-to-mesenchymal transition interactome gene-expression signature is associated with claudin-low and metaplastic breast cancer subtypes**. *Proc Natl Acad Sci U S A* 2010, **107**(35):15449-15454.

16. Urbich C, Kuehbacher A, Dimmeler S: **Role of microRNAs in vascular diseases, inflammation, and angiogenesis**. *Cardiovasc Res* 2008, **79**(4):581-588.
