# Supplementary material for: Integrated genomic analysis of triple-negative breast cancers reveals novel microRNAs associated with clinical and molecular phenotypes and sheds light on the pathways they control
Source: BMC Genomics. 2013 Sep 23;14:643. doi: 10.1186/1471-2164-14-643 (PMC4008358; doi:10.1186/1471-2164-14-643)

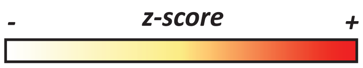

miR me	chromosome	start	stop
miR-214	chr1	172107938	172108047
miR-199a-5p	chr1	172113675	172113784
miR-135b	chr1	205417430	205417526
miR-29c*	chr1	207975197	207975284
miR-29c	chr1	207975197	207975284
miR-664	chr1	220373880	220373961
miR-663b	chr2	133014539	133014653
miR-10b	chr2	177015031	177015140
miR-375	chr2	219866362	219866431
miR-149	chr2	241395418	241395506
miR-425	chr3	49057581	49057667
miR-449a	chr5	54466360	54466450
miR-103	chr5	167987897	167987982
miR-30a*	chr6	72113254	72113324
miR-30a	chr6	72113254	72113324
miR-30c-2*	chr6	72143384	72143455
miR-93	chr7	99691391	99691470
miR-106b*	chr7	99691616	99691697
miR-106b	chr7	99691616	99691697
miR-199b-5p	chr9	131007000	131007109
miR-139-5p	chr11	72326107	72326174
miR-34b*	chr11	111383663	111383746
miR-34c-5p	chr11	111384164	111384240
miR-331-3p	chr12	95702196	95702289
miR-17*	chr13	92002859	92002942
miR-17	chr13	92002859	92002942
miR-18a	chr13	92002997	92003088
miR-19a	chr13	92003145	92003226
miR-20a	chr13	92003319	92003389
miR-19b	chr13	92003446	92003532
miR-19b-1*	chr13	92003446	92003532
miR-92a	chr13	92003568	92003645
miR-342-3p	chr14	100575992	100576090
miR-342-5p	chr14	100575992	100576090
miR-337-3p	chr14	101340830	101340922
miR-184	chr15	79502130	79502213
miR-193b	chr16	14397824	14397906
miR-365	chr16	14403142	14403228
miR-33b	chr17	17717150	17717245
miR-193a-3p	chr17	29887015	29887102
miR-10a	chr17	46657200	46657309
miR-1	chr18	19408965	19409049
let-7c	chr21	17912143	17912236
let-7b	chr22	46509566	46509648
miR-18b	chrX	133304071	133304141
miR-224	chrX	151127050	151127130

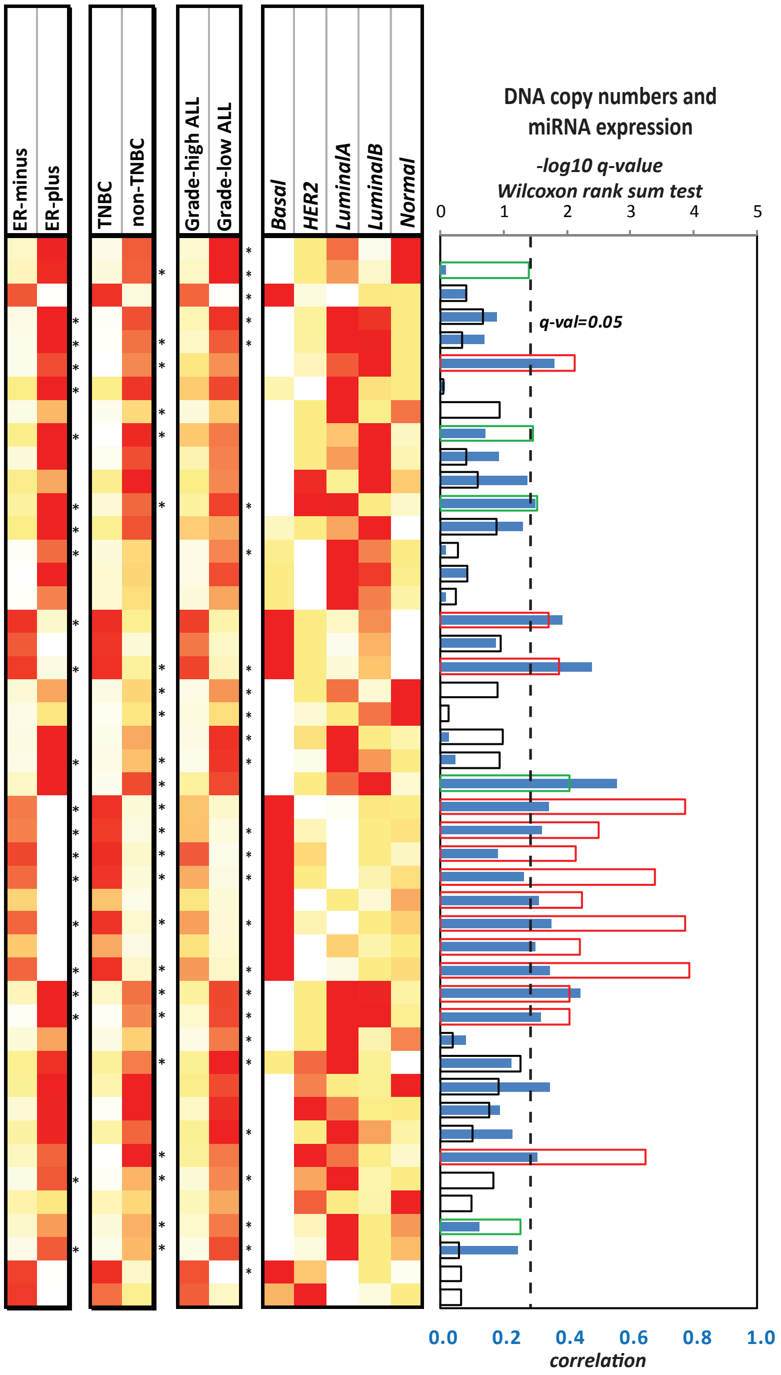

Supplement: Additional file 8 — Details of the analyses carried out for associations with survival and characterization of PAM50 subtype-specific miRNAs. [file 1471-2164-14-643-S8.zip › 4069309791507884_add8/4069309791507884_figS12.pdf]
