# Supplementary material for: Integrated genomic analysis of triple-negative breast cancers reveals novel microRNAs associated with clinical and molecular phenotypes and sheds light on the pathways they control
Source: BMC Genomics. 2013 Sep 23;14:643. doi: 10.1186/1471-2164-14-643 (PMC4008358; doi:10.1186/1471-2164-14-643)
Supplement: Additional file 8 — Details of the analyses carried out for associations with survival and characterization of PAM50 subtype-specific miRNAs. [file 1471-2164-14-643-S8.zip › 4069309791507884_add8/4069309791507884_figS15.pptx]

## Slide 1
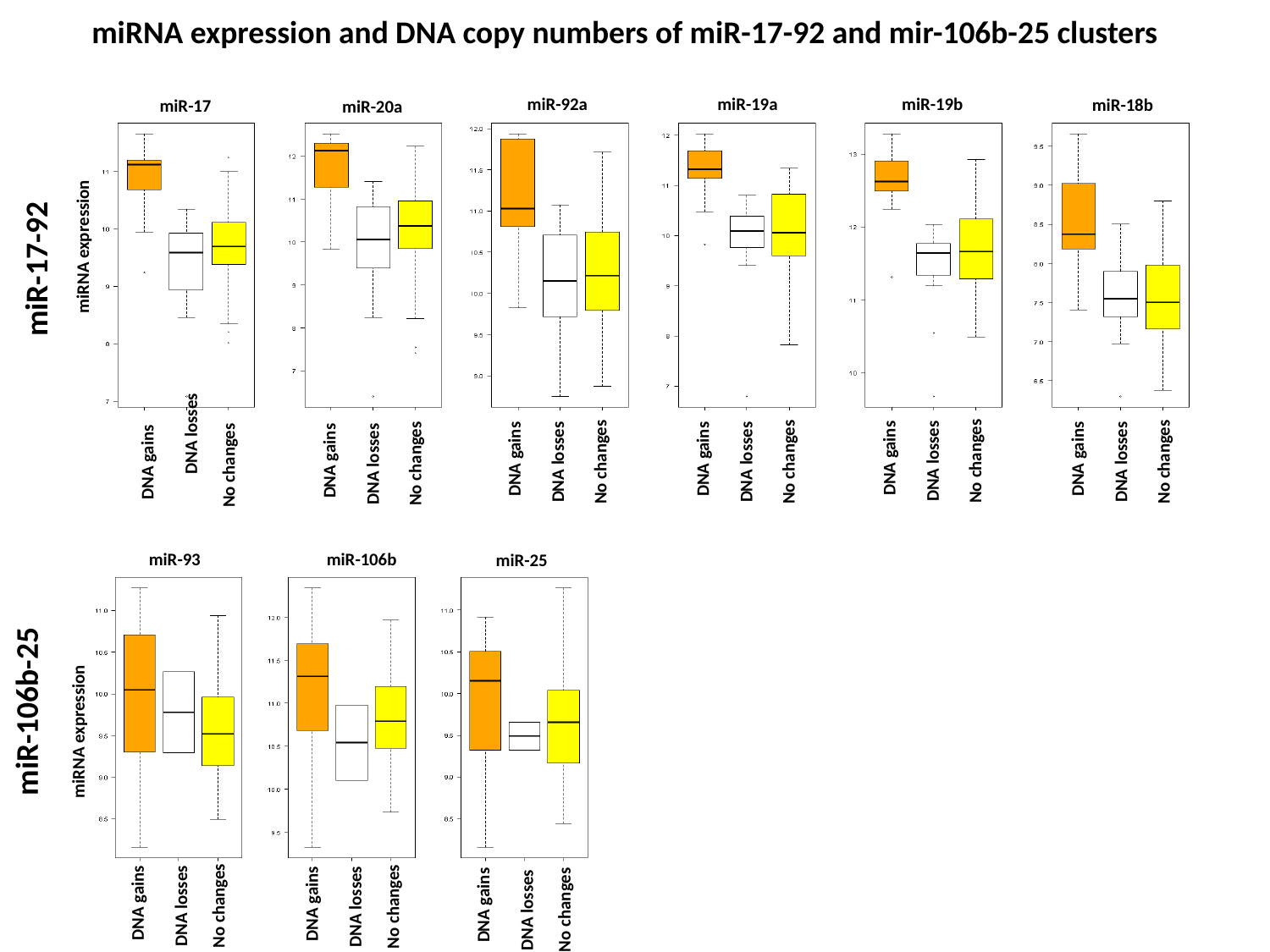

miRNA expression and DNA copy numbers of miR-17-92 and mir-106b-25 clusters
miR-19a
miR-92a
miR-19b
miR-18b
miR-17
miR-20a
miRNA expression
miR-17-92
DNA losses
DNA gains
DNA gains
DNA gains
DNA gains
DNA gains
DNA gains
No changes
No changes
No changes
No changes
DNA losses
DNA losses
No changes
DNA losses
DNA losses
No changes
DNA losses
miR-93
miR-106b
miR-25
miR-106b-25
miRNA expression
DNA gains
DNA gains
DNA gains
No changes
No changes
DNA losses
DNA losses
No changes
DNA losses
