# Supplementary material for: Integrated genomic analysis of triple-negative breast cancers reveals novel microRNAs associated with clinical and molecular phenotypes and sheds light on the pathways they control
Source: BMC Genomics. 2013 Sep 23;14:643. doi: 10.1186/1471-2164-14-643 (PMC4008358; doi:10.1186/1471-2164-14-643)
Supplement: Additional file 8 — Details of the analyses carried out for associations with survival and characterization of PAM50 subtype-specific miRNAs. [file 1471-2164-14-643-S8.zip › 4069309791507884_add8/4069309791507884_figS6.pptx]

## Slide 1
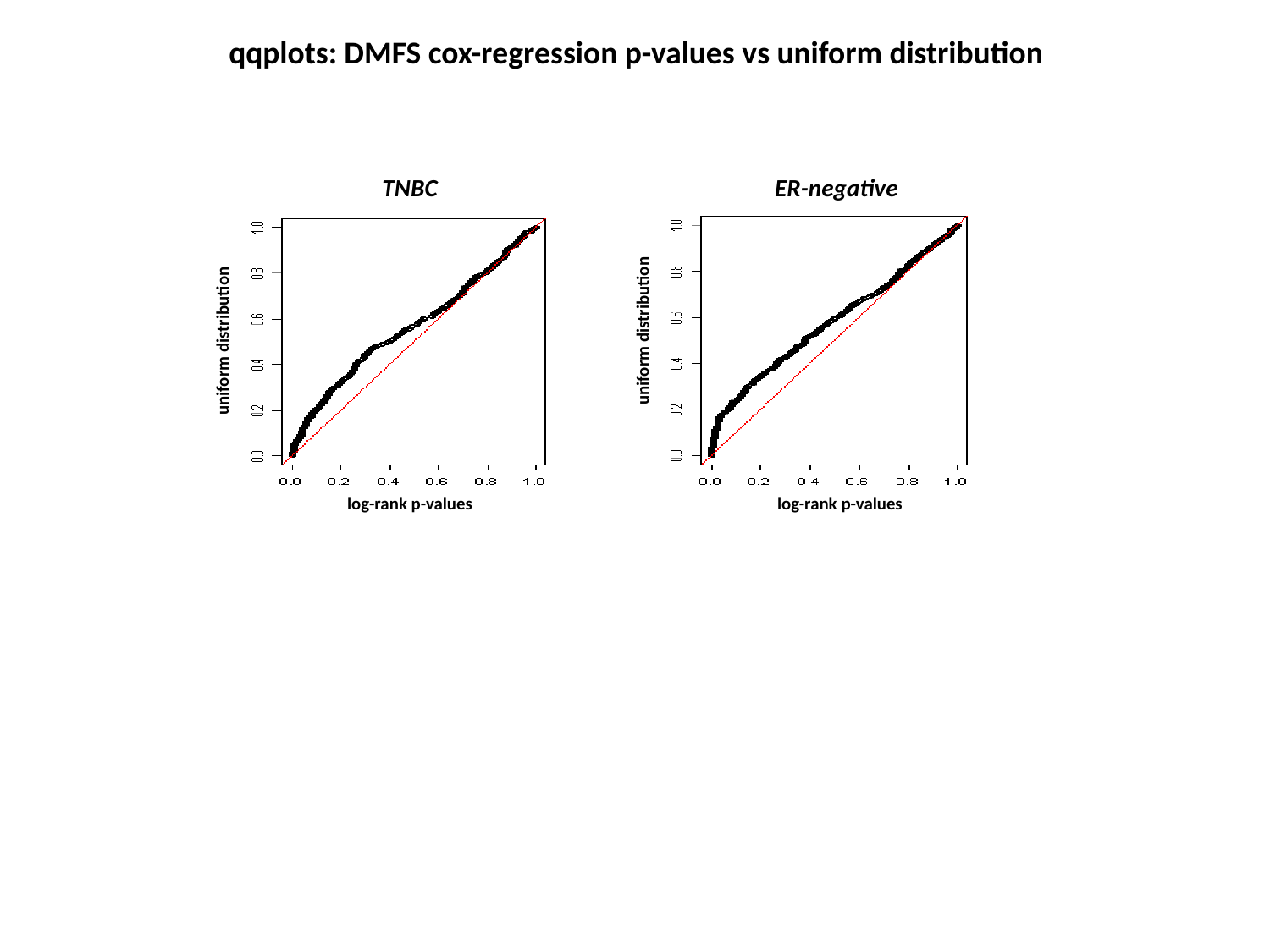

qqplots: DMFS cox-regression p-values vs uniform distribution
TNBC
ER-negative
uniform distribution
uniform distribution
log-rank p-values
log-rank p-values
