# Supplementary material for: Integrated genomic analysis of triple-negative breast cancers reveals novel microRNAs associated with clinical and molecular phenotypes and sheds light on the pathways they control
Source: BMC Genomics. 2013 Sep 23;14:643. doi: 10.1186/1471-2164-14-643 (PMC4008358; doi:10.1186/1471-2164-14-643)
Supplement: Additional file 8 — Details of the analyses carried out for associations with survival and characterization of PAM50 subtype-specific miRNAs. [file 1471-2164-14-643-S8.zip › 4069309791507884_add8/Additional file 8 captions.doc]

**Figure S5** Forest plot of Hazard ratios of the 14 miRNAs collectively identified to be associated with DMFS and/or BCSS clinical follow-ups. Log2 proportional hazard ratios and 95th confidence intervals are represented. **Figure S6** Quantile-quantile plots comparing the distribution of the p-values from log-rank tests computed from different DMFS Cox regression models (Y-axis), and the uniform distribution (X-axis). **Figure S7** Distribution of miRNA p-values for association with breast cancer specific survival, when miRNAs were evaluated together with histopathological covariates. Each box corresponds to a different Cox-regression model, as illustrated in the legend. **Figure S8** Hazard ratios of 6 miRNAs retaining their association with prognosis (DMFS and/or BCSS) when evaluated together with node positivity and tumour size in a multivariate Cox-regression model. ”N”,”S”,”L” stand respectively for Grading, Node positivity, tumour size, percentage of lymphocytic infiltration. Log2 proportional hazard ratios and 95th confidence intervals are represented. **Figure S9** Hazard ratios of the 14 miRNAs collectively identified to be associated with DMFS and/or BCSS clinical follow-ups. Log2 proportional hazard ratios and 95th confidence intervals from the analysis of ER-negative samples are represented, across the 3 different data sets (“Guy’s” label refers to the data set we have used). **Figure S10** Hazard ratios of the 14 miRNAs collectively identified to be associated with DMFS and/or BCSS clinical follow-ups. Log2 proportional hazard ratios and 95th confidence intervals from the analysis of TNBC samples are represented, across the 3 different data sets (“Guy’s” label refers to the data set we have used). **Figure S11** Distribution of miRNA and mRNA p-values for association with breast cancer specific survival in TNBC and ER-negative samples. **Figure S12** miRNAs differentially expressed between different intrinsic subtypes are reported (p-value<0.0001, FDR q-value <0.001). Left Panel: Heatmap representing the averaged z-scores in different tumour groups. Differential expressions between group-pairs are marked with an asterisk in the right margins (FDR q-value < 0.01). miRNAs members of the miR-17-92 and miR-106b-25 clusters are respectively highlighted in purple and red font colours. Right Panel: Association between miRNA expression and DNA gains/losses. Filled blue bars represent Spearman correlation values. Red and green shaded bars represent the -log10 q-value of the Wilcoxon test comparing respectively tumours with DNA gains and losses to tumours without DNA changes. miR-106b-25^(*)^ and miR-17-92 cluster members are respectively highlighted in red and purple. ^(*)^miR-25 (also member of the miR-106b-25 cluster) is not included in the list as it did not pass the stringent cut-offs used for the selection, despite having a significant over-expression in basal-like breast cancers (FDR=0.005). **Figure S13** miR-17-92 and miR-106b-25 miRNA clusters. a) Structure and location of the two miRNA clusters within intronic regions their respective host genes b) Scatter plots showing of the expression levels of the miRNA clusters (expressed as an average of the miRNA cluster members) and their respective host genes. **Figure S14** Frequencies of DNA gains (Y axis, green colour) and losses (Y axis, red colour) along the genome sequence (X axis) of chromosomes 7 and 13 are represented. Chromosomal locations of miR-17-92 and miR-106b-25 clusters are highlighted. Data refer to the selected sub-cohort of basal-like tumours. **Figure S15** Box-plots showing the expression levels of miRNAs of the miR-106b-25 and miR-17-92 clusters, across the entire panel of tumours, grouped according to DNA gains (orange), losses (white), no changes (yellow) of the respective loci. **Figure S16** Comparative analysis between our study and Blenkiron's study [6] of subtype-specific miRNAs. X axis: FDR threshold used to select subtype specific miRNAs in our study. Right Y-axis (in red): overlap obtained with Blenkiron's list. Left Y-axis (in blue): -log10 Fisher-test p-value of the overlap between our and Blenkiron's lists. **Figure S17** Scatter plot showing the expression levels of the miR-17 and MYC gene across all tumours.
